# Unveiling degradation mechanism of PAHs by a *Sphingobium* strain from a microbial consortium

**DOI:** 10.1002/mlf2.12032

**Published:** 2022-07-25

**Authors:** Lige Zhang, Huan Liu, Junbiao Dai, Ping Xu, Hongzhi Tang

**Affiliations:** ^1^ State Key Laboratory of Microbial Metabolism, Joint International Research Laboratory of Metabolic and Developmental Sciences, and School of Life Sciences and Biotechnology Shanghai Jiao Tong University Shanghai China; ^2^ CAS Key Laboratory of Quantitative Engineering Biology, Guangdong Provincial Key Laboratory of Synthetic Genomics and Shenzhen Key Laboratory of Synthetic Genomics, Shenzhen Institute of Synthetic Biology, Shenzhen Institutes of Advanced Technology Chinese Academy of Sciences Shenzhen China

**Keywords:** metagenomic binning analysis, P450, polycyclic aromatic hydrocarbons, ring‐hydroxylating dioxygenase, *Sphingobium*

## Abstract

Polycyclic aromatic hydrocarbons (PAHs) are a class of persistent pollutants with adverse biological effects and pose a serious threat to ecological environments and human health. The previously isolated phenanthrene‐degrading bacterial consortium (PDMC) consists of the genera *Sphingobium* and *Pseudomonas* and can degrade a wide range of PAHs. To identify the degradation mechanism of PAHs in the consortium PDMC, metagenomic binning was conducted and a *Sphingomonadales* assembly genome with 100% completeness was obtained. Additionally, *Sphingobium* sp. SHPJ‐2, an efficient degrader of PAHs, was successfully isolated from the consortium PDMC. Strain SHPJ‐2 has powerful degrading abilities and various degradation pathways of high‐molecular‐weight PAHs, including fluoranthene, pyrene, benzo[a]anthracene, and chrysene. Two ring‐hydroxylating dioxygenases, five cytochrome P450s, and a pair of electron transfer chains associated with PAH degradation in strain SHPJ‐2, which share 83.0%–99.0% similarity with their corresponding homologous proteins, were identified by a combination of *Sphingomonadales* assembly genome annotation, reverse‐transcription quantitative polymerase chain reaction and heterologous expression. Furthermore, when coexpressed in *Escherichia coli* BL21(DE3) with the appropriate electron transfer chain, PhnA1B1 could effectively degrade chrysene and benzo[a]anthracene, while PhnA2B2 degrade fluoranthene. Altogether, these results provide a comprehensive assessment of strain SHPJ‐2 and contribute to a better understanding of the molecular mechanism responsible for the PAH degradation.

## Impact statement

Polycyclic aromatic hydrocarbons (PAHs) are ubiquitous and have attracted widespread attention due to their adverse effects. Several bacteria acquired the capability to degrade PAHs, with *Sphingobium* strains being the most dominant genus. However, the degradation mechanisms in *Sphingobium* strains that degrade high‐molecular‐weight PAHs are poorly understood. Here, *Sphingobium* sp. strain SHPJ‐2, a highly efficient PAH degrader in the phenanthrene‐degrading bacterial consortium, was identified and characterized. In addition, several ring‐hydroxylating dioxygenases and P450s which can degrade high‐molecular‐weight PAHs were also identified. Our findings reveal that strain SHPJ‐2 has efficient PAHs‐degrading genetic resources and could help unveil the degradation mechanisms related to PAH degradation in Sphingomonads.

## INTRODUCTION

Polycyclic aromatic hydrocarbons (PAHs), with two or more fused aromatic rings, are primarily produced by the incomplete combustion of organic compounds (such as fossil fuels and municipal wastes) under high‐temperature conditions^1^. Widespread anthropogenic activities make PAHs ubiquitous in various ecosystems, including soil, sediment, and the atmosphere. PAHs have attracted worldwide attention due to their genotoxicity, carcinogenicity, teratogenicity, mutagenicity, and bioaccumulation^2^. Of the hundreds of known PAHs, 16 have been designated as High Priority Pollutants by the Environmental Protection Agency^3^.

Microbial degradation relies on the powerful metabolic detoxification ability of naturally degrading bacteria. Its advantages of mild reaction conditions, high‐degradation efficiency, and low cost have made it the most effective method for environmental remediation^4^. The earliest reports of aerobic PAH degradation by bacteria date back to the 1940s^5^. Since then, many PAH‐degrading strains have been identified in different genera, which are mainly distributed in *Pseudomonas*, *Sphingomonas*, *Sphingobium*, *Mycobacterium*, and *Rhodococcus* ^6^. Among them, sphingomonad members (including *Sphingomonas*, *Sphingobium, Sphingopyxis*, and *Novosphingobium*) are particularly important because of their ability to degrade a wide range of recalcitrant natural and anthropogenic aromatic compounds, especially PAHs^7^. This extraordinary metabolic property can be attributed to their multiple degradation genes and unique gene arrangement^8^.

Over the past few decades, the functional enzymes related to PAH degradation in bacteria have been extensively investigated^9^. In particular, ring‐hydroxylating dioxygenases (RHDs), which are members of a Rieske nonheme iron oxygenase family, typically initiate the oxidation of aromatic compounds to form *cis*‐dihydrodiols^10^. RHDs are multicomponent enzymes consisting of two or three proteins, including an electron transfer chain and a terminal dioxygenase component. The terminal dioxygenase component is generally a homologous (α_*n*_) or heterodimeric oligomer (α_*n*_β_*n*_). The conserved α‐subunit is closely related to substrate recognition and catalysis and is responsible for transferring electrons to the oxygen atom, which has made it popular with researchers^11^. In addition, cytochrome P450 monooxygenases (P450s) have the potential to catalyze a wide range of oxidation reactions (e.g., hydroxylation, epoxidation, and dealkylation) and can transform PAHs into hydroxylated PAHs^12^, ^13^. A significant amount of RHDs or P450s have been reported to degrade low‐molecular‐weight PAHs (LMW‐PAHs)^14^, ^15^. However, knowledge about the functional enzymes capable of degrading high‐molecular‐weight PAHs (HMW‐PAHs) remains still limited compared to LMW‐PAHs. The few reported RHDs that can degrade HMW‐PAHs usually share relatively low amino acid similarity and have unique catalytic specificities for PAHs, such as the Nid^16^, ^17^, Phn^18^, ^19^, and Pdo systems^20^. Therefore, identifying and characterizing more functional enzymes that degrade HMW‐PAHs is important to clarify the bacterial degradation mechanisms.

In our previous study, the efficient phenanthrene (PHE)‐degrading bacterial consortium PDMC, consisting of the genera *Sphingobium* (58.57%–72.40%) and *Pseudomonas* (25.93%–39.75%), was obtained and characterized. The versatile PDMC consortium has multisubstrate degradation capabilities, including PHE, naphthalene (NAP), acenaphthene (ACE), fluorene (FLN), anthracene (ANT), fluoranthene (FLU), benzo[a]anthracene (BaA), dibenzothiophene (DBT), dibenzofuran (DBF), carbazole (CA), and indole^21^. However, the functional genes of the consortium PDMC have not yet been characterized. Herein, we first performed metagenomic sequencing and binning analysis and then determined the dominant degradation genera based on the number of degradation genes in the resulting genome bins. The PAHs‐degrading strains from consortium PDMC were then isolated and characterized. Several potentially efficient PAHs‐degrading genes encoding RHDs or P450s in the isolated strain were identified in combination with the metagenomic binning analysis. In addition, the substrate specificities of two important RHDs were separately analyzed with 12 representative PAHs substrates. Since genetic information on the degradation of HMW‐PAHs is limited, the in‐depth study allows us to better understand and unveil the PAH degradation mechanism, which lays an important foundation for subsequent genetic utilization and bioremediation.

## RESULTS AND DISCUSSION

### Bin9 has abundant xenobiotics biodegradation‐related genes

Metagenomic sequencing of consortium PDMC by the Illumina NovaSeq platform yielded 142,368,368 paired‐end reads and 21,355,255,200 bp bases. After applying a quality filter, a total of 21,233,932,858 bp of valid sequences with 63.57% guanine–cytosine (GC) content were obtained, and the percentage of bases with sequencing accuracies exceeding 99% (Q20) and 99.9% (Q30) were 97.62% and 93.32%, respectively. Assembly using IDBA‐UD constructed 9980 contigs with an average length of 3835 bp and an N50 of 103,950 bp (Table S1).

Binning with MetaWRAP generated seven genome bins with completeness scores of >90% and <5% level of contamination. These bins ranged from 3.9 to 5.7 Mbp, and their GC content varied from 37% to 74%. Of them, bin6 and bin9 had the highest integrity scores (100%), with contamination rates of 0% and 2.161%, respectively. Taxonomic annotations were distinct among these seven genome bins. Bin5 and bin6 belonged to the bacterial phylum *Bacteroidetes* and class *Alphaproteobacteria*, respectively; bin2 and bin7 both belonged to the order *Actinomycetes*, while bin8 and bin9 belonged to the orders *Pseudomonadales* and *Sphingomonadales*, respectively; and bin4 was annotated to the species *Rhodobacteraceae* (Table 1).

**Table 1:** The evaluation results of high‐quality MAGs from consortium PDMC.

Bin	Completeness (%)	Contamination (%)	GC (%)	Lineage	N50 (bp)	Size (bp)
Bin2	92.58	2.023	74.4	*Actinomycetales*	7390	4,087,014
Bin4	99.62	0.666	62.3	*Rhodobacteraceae*	193,502	4,087,468
Bin5	99.36	0.952	37.2	*Bacteroidetes*	92,177	4,506,802
Bin6	100	0	56.1	*Alphaproteobacteria*	448,205	5,094,743
Bin7	99.49	0	68.3	*Actinomycetales*	233,046	3,904,791
Bin8	99.83	0.408	63.2	*Pseudomonadales*	239,276	5,714,656
Bin9	100	2.161	64.5	*Sphingomonadales*	129,435	5,212,268

**Figure 1:**
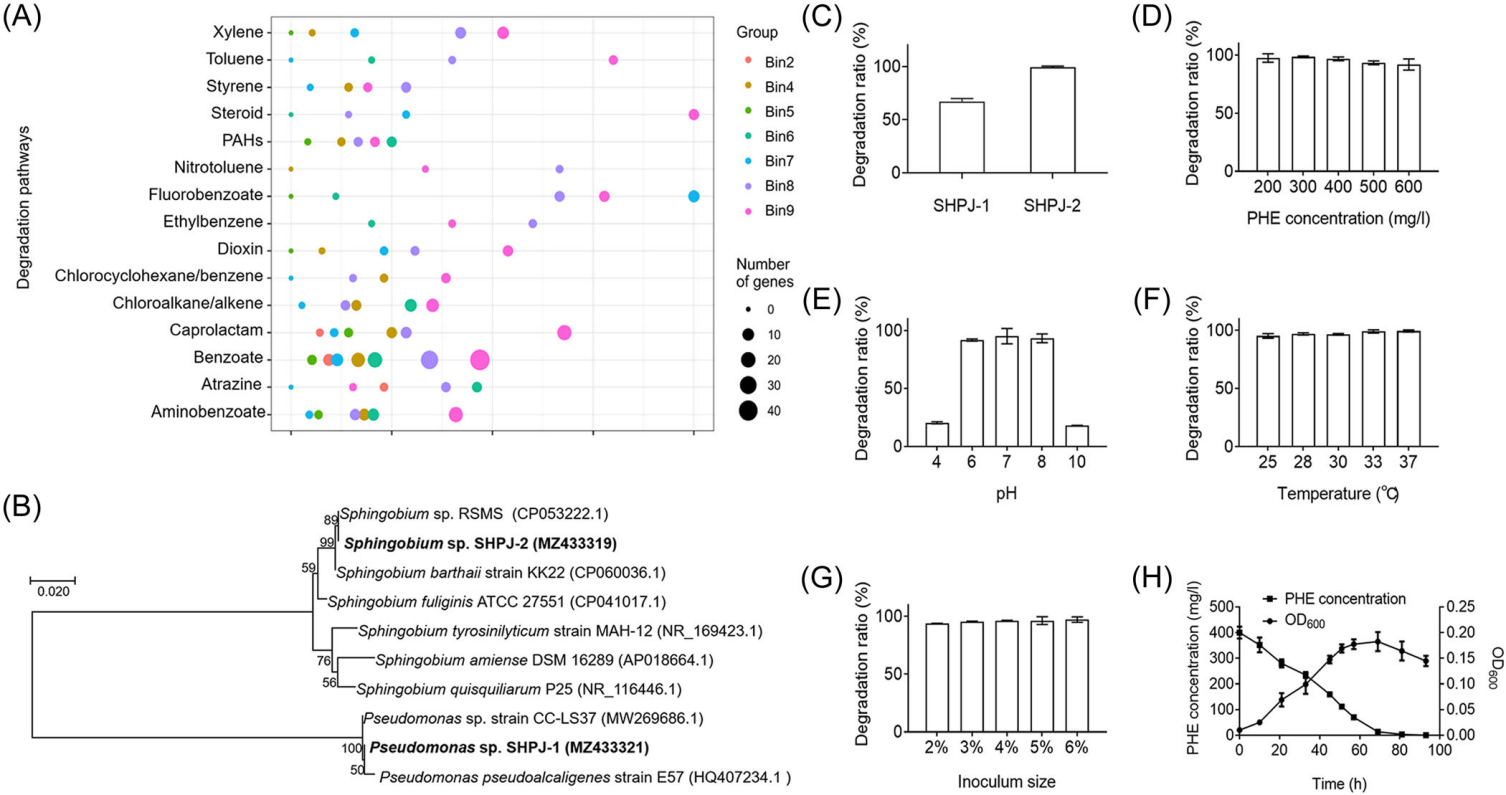
Gene prediction of consortium PDMC and PHE degradation characteristics of PHE degraders. (A) The genes involved in the metabolism of xenobiotics in each MAG of consortium PDMC were predicted by KAAS. The *X*‐axis is the ratio of the number of genes predicted in each MAG to the total number of genes predicted in the PDMC consortium, *Y*‐axis is the type of xenobiotics biodegradation and metabolism, and the size of the bubble indicates the number of genes. (B) Phylogenetic tree based on comparison of 16S ribosomal RNA of strain SHPJ‐1 and SHPJ‐2 with similar sequences^23^, ^24^, ^25^, ^26^, ^27^, ^28^. The tree was constructed by MEGA X using the neighbor‐joining method with bootstrap analysis of 1000 replicates. Accession numbers are from the NCBI database. (C–H) The PHE degradation activity of strains isolated from consortium PDMC. The degradation ratios of PHE by *Pseudomonas* sp. SHPJ‐1 and *Sphingobium* sp. SHPJ‐2 within 72 h (C). The effect of PHE concentration (D), pH (E), temperature (F), and inoculum size (G) on the degradation of strain SHPJ‐2. Growth and degradation curve of strain SHPJ‐2 in MSM containing PHE under optimal conditions (H). MAG, metagenomic assembled genome; MSM, mineral salt medium; PDMC, phenanthrene‐degrading bacterial consortium; PHE, phenanthrene.

To understand the functional capacities of the bacterial consortium PDMC and seven genome bins, we used KEGG Automatic Annotation Server ver. 2.1 (KAAS) based on the predicted open reading frames (ORFs) to perform functional annotations. Notably, xenobiotics contain PAHs and several aromatic compounds, and some PAH degradation genes are known to degrade aromatic compounds, such as *nahA* ^22^ and *nidA3B3* ^17^. Therefore, we predicted the pathways and genes related to xenobiotics degradation. In total, consortium PDMC had at least 15 pathways related to xenobiotic biodegradation and metabolism, and all seven bins contained several genes associated with these pathways (Figure 1A). Of them, bin8 and bin9 contained the most abundant xenobiotics biodegradation‐related genes. Specifically, bin8 contained 33, 2, 8, 3, 4, 4, and 1 gene related to benzoate, toluene, xylene, ethylbenzene, dioxins, PAHs, and steroid metabolism, respectively. Similarly, bin9 contained 45, 4, 10, 2, 7, 5, and 7 genes associated with these seven metabolic pathways, accounting for 46.9%, 80.0%, 52.6%, 40.1%, 53.8%, 20.8%, and 100% of the total associated genes in consortium PDMC, respectively. These results demonstrate that bin9, annotated as the order *Sphingomonadales*, contained the most abundant genes for xenobiotics metabolism. This indicates that it could play a dominant role in the PAH and heterocyclic degradation of consortium PDMC.

### Strain SHPJ‐2 has efficient degradation capability

Two PHE degraders, SHPJ‐1 and SHPJ‐2, were isolated from consortium PDMC after spreading sequential multiple plates. Both strains grew in the basic mineral salt medium (MSM) containing PHE as the sole carbon source. Neighbor‐joining phylogenetic analysis revealed that the 16S ribosomal RNA (rRNA) gene sequences of SHPJ‐1 and SHPJ‐2 had the highest similarity to the *Pseudomonas* sp. strain CC‐LS37 (100%, MW269686.1) and *Sphingobium* sp. RSMS (99.0%, CP053222.1), respectively (Figure 1B). When cultured in MSM with 400 mg/L PHE as the sole carbon source, the degradation ratios of *Pseudomonas* sp. SHPJ‐1 and *Sphingobium* sp. SHPJ‐2 were 67.18% and 98.68% at 72 h, respectively (Figure 1C). As a result, *Sphingobium* sp. SHPJ‐2 was selected for follow‐up studies due to its higher capacity for PHE degradation.

The degradation conditions of strain SHPJ‐2 were optimized under a single variable, including PHE concentration, pH values, culture temperature and inoculum size. As shown in Figure 1D–G, the SHPJ‐2 strain had robust degradation capacity under various conditions, including 200–600 mg/l initial PHE concentration, pH 6–8, 25–37°C, or 2%–6% of inoculum levels. Compared with other reported PHE‐degrading strains, strain SHPJ‐2 had a significant advantage in tolerance and degradation of higher concentrations of PHE (400–600 mg/l)^29^, ^30^, ^31^. Additionally, the PHE degradation ability of strain SHPJ‐2 did not change significantly at temperatures ranging from 25°C to 37°C within 72 h, indicating that the strain can adapt to a wide range of temperatures. Optimal degradation was observed at 400 mg/l PHE as the sole carbon source, pH 7, 5% inoculum size, and 30°C incubation. Under these conditions, strain SHPJ‐2 entered the logarithmic growth period at 10 h and the growth rate gradually increased, reaching a maximum OD_600_ of 0.18 at 69 h. The PHE concentration also decreased as the strain grew, and 97.5% of PHE was degraded at 69 h with an average degradation efficiency of approximately 5.65 mg/l/h (Figure 1H). This exceeded most known PHE‐degrading strains, including *Pseudomonas* sp. USTB‐RU^32^, *Rhodococcus wratislaviensis* strain 9^33^, *Mycobacterium* sp. WY10^34^, *Klebsiella* sp. PD3^35^, and *Rhodococcus pyridinivorans* DTU‐7P^36^.

In the resting cell reactions, 400 mg/L PHE was completely degraded by PHE‐grown SHPJ‐2 but not by glycerol‐grown bacteria within 12 h (Figure S1). This indicates that strain SHPJ‐2 could be induced to degrade PHE and be used to determine changes in the transcript levels of the relevant degradation genes when PHE or glycerol is the sole carbon source.

### Strain SHPJ‐2 has a broad substrate spectrum

**Figure 2:**
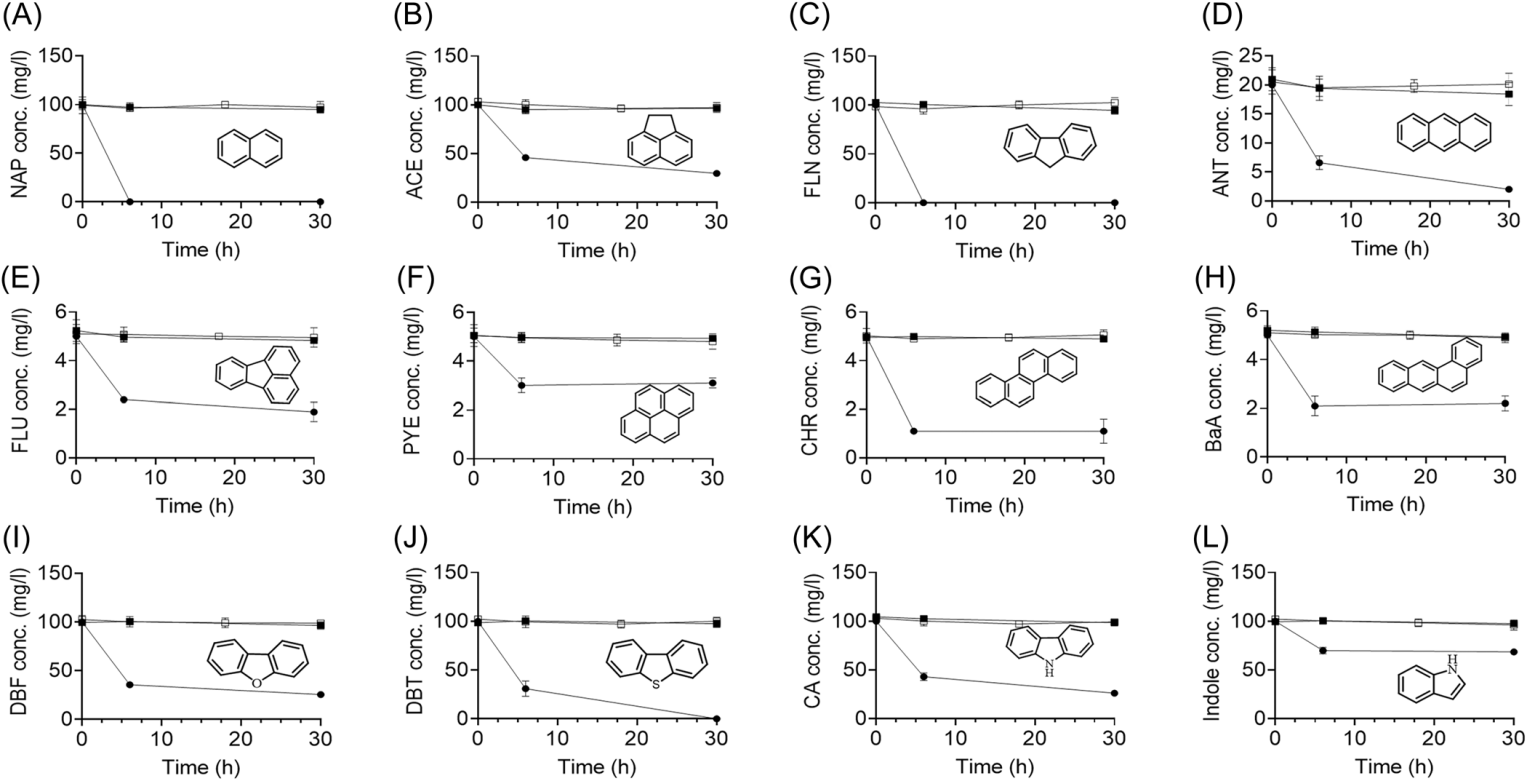
Degradation of polycyclic aromatic hydrocarbons and heterocyclic derivatives using strain SHPJ‐2 resting cells. (A) NAP (100 mg/l); (B) ACE (100 mg/l); (C) FLN (100 mg/l); (D) ANT (20 mg/l); (E) FLU (5 mg/l); (F) PYE (5 mg/l); (G) CHR (5 mg/l); (H) BaA (5 mg/l); (I) DBF (100 mg/l); (J) DBT (100 mg/l); (K) CA (100 mg/l); (L) indole (100 mg/l). Black box (■), white box (□), and circle (●) indicate the blank control group, inactive cell control group, and experimental group, respectively. The concentration in parentheses is starting concentration. ACE, acenaphthene; ANT, anthracene; BaA, benzo[a]anthracene; CA, carbazole; CHR, chrysene; DBF, dibenzofuran; DBT, dibenzothiophene; FLN, fluorene; FLU, fluoranthene; NAP, naphthalene; PYE, pyrene.

PAHs‐degrading strains are not usually limited to degrading a specific substrate but can degrade multiple PAHs or heterocyclic derivatives. As shown in Figure 2, the resting cells of the SHPJ‐2 strain achieved 100% degradation of NAP (Figure 2A) and FLN (Figure 2C) within 6 h. The highest degradation ratios were reached at 30 h for ACE (Figure 2B), ANT (Figure 2D), FLU (Figure 2E), DBF (Figure 2I), DBT (Figure 2J), and CA (Figure 2K), with 70.3%, 91.3%, 62.2%, 74.5%, 100%, and 74.4%, respectively. In addition, the SHPJ‐2 strain degraded pyrene (PYE, Figure 2F), chrysene (CHR, Figure 2G), BaA (Figure 2H) and indole (Figure 2L) to some extent within 6 h, with degradation ratios of 39.0%, 78.0%, 55.3%, and 31.5%, respectively. Therefore, we concluded that strain SHPJ‐2 has a high degradation capability and a broad substrate spectrum with potential applications in the bioremediation of mixed PAHs‐contaminated environments.

### Strain SHPJ‐2 has multiple PAH degradation pathways

**Figure 3:**
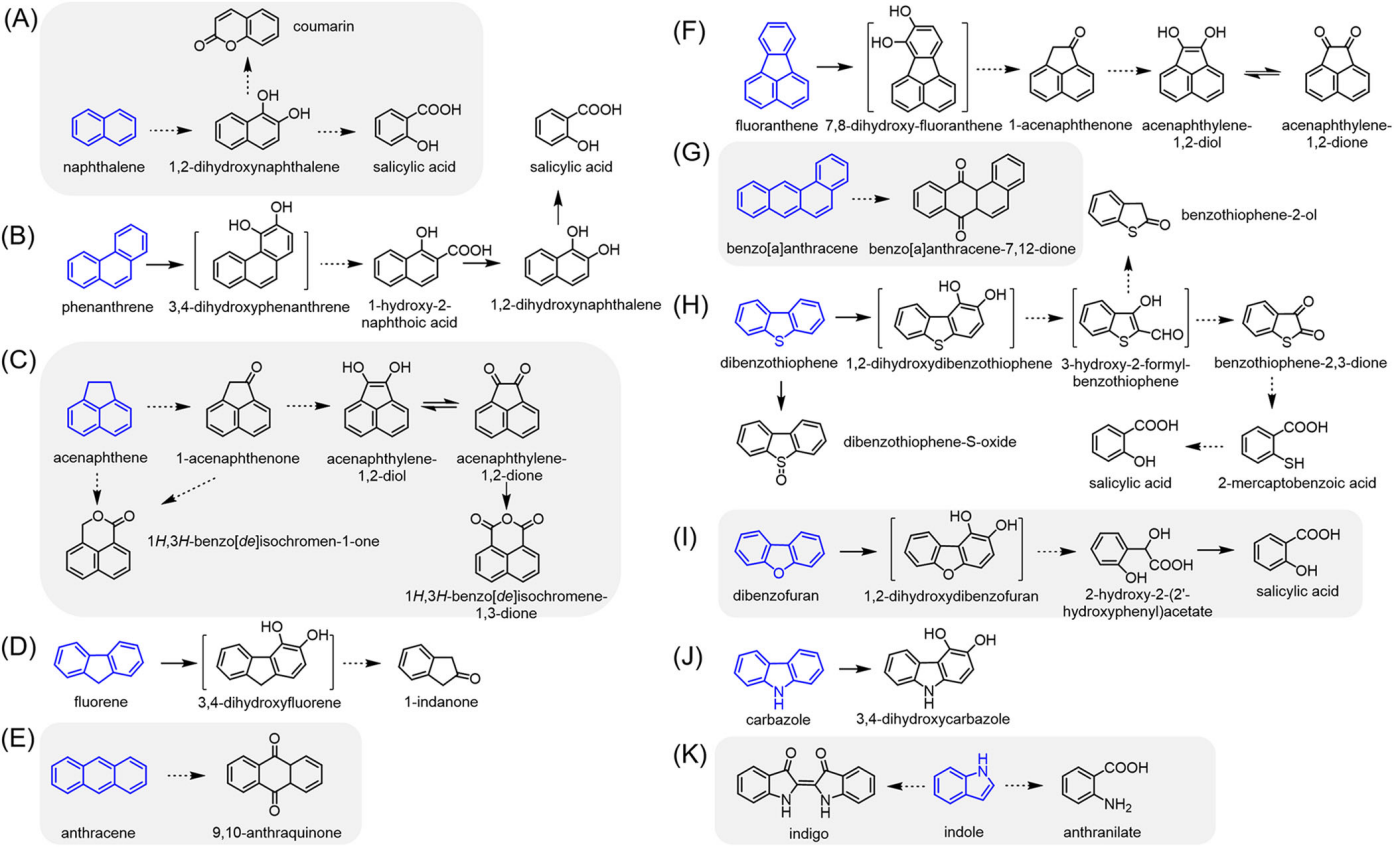
Proposed pathways for the metabolism of polycyclic aromatic hydrocarbons and heterocyclic derivatives by strain SHPJ‐2. (A) NAP; (B) PHE; (C) ACE; (D) FLN; (E) ANT; (F) FLU; (G) BaA; (H) DBT; (I) DBF; (J) CA; (K) indole. Blue is the substrate; products in brackets were not detected. The solid and dotted arrows represent one‐step or multistep reactions, respectively.

The intermediate metabolites found in PAH and heterocyclic derivative degradation by strain SHPJ‐2 were detected by gas chromatography‐mass spectrometry (GC‐MS) and identified by comparing with the National Institute of Standards and Technology (NIST) MS library or MS profiles of the standard compounds. Specific information relating to these intermediates is summarized in Table S2. The PAH degradation pathways by strain SHPJ‐2 were proposed based on detected intermediate metabolites and reported studies (Figure 3).

GC‐MS results of NAP samples revealed five major intermediate metabolites, namely, 1‐naphthol (11.594 min), 2‐naphthol (11.618 min), and coumarin (10.815 min), as well as tetramethylsilane (TMS)‐derived salicylic acid (11.577 min) and 1,2‐dihydroxynaphthalene (14.251 min). Of them, 1‐naphthol and 2‐naphthol can be catalyzed by P450s or produced by the spontaneous dehydration of the unstable product *cis*‐1,2‐dihydroxy‐1,2‐dihydroxynaphthalene generated during NAP degradation^37^. As for the PHE samples, we detected 1‐hydroxy‐2‐naphthoic acid (14.875 min), 1‐naphthol (11.594 min), and TMS‐derived salicylic acid (11.577 min). All three products are common metabolites prevalent in the PHE degradation pathway and can be detected in the bacterial consortium PDMC^21^. Therefore, we hypothesize that strain SHPJ‐2 degraded NAP and PHE at the C‐1,2 and C‐3,4 positions, respectively, which eventually cleave the ring to produce salicylic acid. Since strain SHPJ‐2 uses PHE as the sole carbon growth source, the downstream product (salicylic acid) can enter the TCA cycle and achieve complete PHE mineralization. Proposed pathways for the degradation of the NAP and PHE by strain SHPJ‐2 according to the previous studies are shown in Figure 3A,B, respectively^37^.

Five intermediate metabolites, namely, 1‐acenaphthenone (13.541 min), 1*H*,3*H*‐benzo[de]isochromen‐1‐one (14.817 min), acenaphthylene‐1,2‐dione (15.493 min), 1*H*,3*H*‐benzo[de]isochromene‐1,3‐one (16.838 min), and TMS‐derived 1,2‐dihydroxyacenaphthylene (16.711 min), were detected in the ACE samples (Figure 3C). We detected only one intermediate in the FLN samples: 1‐indanone with a retention time of 8.961 min, an intermediate metabolite in the FLN C‐3,4 position metabolic pathway (Figure 3D)^38^. Similarly, 9,10‐anthraquinone was the only metabolite detected in ANT metabolism samples, usually the signature intermediate metabolite and end‐product of the ANT C‐9,10 position metabolic pathway (Figure 3E)^39^. In the FLU samples, we identified three intermediate metabolites as 1‐acenaphthenone (13.541 min), acenaphthylene‐1,2‐dione (15.493 min), and TMS‐derived 1,2‐dihydroxyacenaphthenone (16.711 min); all of these were also detected in the ACE samples. Combined with previous studies^37^, it is clear that the SHPJ‐2 strain can initiate dioxygenation at the FLU C‐7,8 position and merge with the downstream metabolic pathway of ACE after the ring‐cleavage reaction (Figure 3F).

PYE, CHR, and BaA are HMW‐PAHs with stable structures and high toxicity. We detected only one intermediate metabolite in the BaA sample, BaA‐7,12‐dione, with a retention time of 21.493 min. It was generated after initiating dioxygenated metabolism at the C‐7,12 position (Figure 3G)^40^. However, we did not detect any metabolic intermediates of PYE and CHR. This could be due to the low initial concentration of the substrate (5 mg/l) and its low biodegradation ratio (39.0%–78.0%), making them difficult to detect. While no PYE‐related metabolic intermediates were detected in the resting cell reaction of strain SHPJ‐2, we detected the corresponding monohydroxy products in the subsequent substrate specificity assays with PhnA1B1 and PhnA2B2, confirming the ability of strain SHPJ‐2 to convert PYE^41^.

### Proposed heterocyclic derivative degradation pathways of strain SHPJ‐2

As for the degradation of heterocyclic derivatives, we detected benzothiophene‐2‐ol (10.514 min), benzothiophene‐2,3‐dione (11.438 min), dibenzothiophene‐*S*‐oxide (17.058 min), TMS‐derivatized salicylic acid (11.577 min) and TMS‐derivatized 2‐mercaptobenzoic acid (13.558 min) in the DBT samples. We hypothesized that strain SHPJ‐2 could degrade DBT through the Kodama pathway and the “4S” pathway based on previous studies (Figure 3H). In the Kodama pathway, DBT can successively generate undetectable 1,2‐dihydroxydibenzothiophene and 3‐hydroxy‐2‐formyl‐benzothiophene. The latter was then oxidized to benzothiophene‐2‐ol and benzothiophene‐2,3‐dione^42^, after which benzothiophene‐2,3‐dione was successively converted to 2‐mercaptobenzoic acid and salicylic acid. While in the “4S” pathway, strain SHPJ‐2 only transformed DBT into dibenzothiophene‐*S*‐oxide via one‐step monooxygenation^43^. In the TMS‐derived DBF samples, we detected salicylic acid (11.577 min) and 2‐hydroxy‐2‐(2ʹ‐hydroxyphenyl)acetate (13.148 min), which were the intermediate products of DBF degradation via the lateral dioxygenation pathway^44^. For the CA samples, TMS‐derived 3‐hydrocarbazole, which appeared at 16.007 min, was the only intermediate metabolite detected. This substance was generated for reasons similar to those described above for 1‐naphthol and 2‐naphthol. It can be generated by either P450s catalysis or by spontaneous dehydration of the unstable intermediate metabolite 3,4‐carbazole‐dihydrodiol. The latter is the main reason for the formation of 3‐hydrocarbazole in most bacteria^45^. Therefore, we speculated that the SHPJ‐2 strain degraded DBF and CA via the lateral dioxygenation pathways, which begin at the C‐1,2 and C‐3,4 positions, respectively (Figure 3I,J)^46^, ^47^. Blue particles visible to the naked eye appeared in the indole medium, identified as indigo. Additionally, a peak at 11.259 min in the derivatized sample was anthranilate. Both are common degradation products of indole (Figure 3K)^35^.

### Identification of possible RHDs and P450s of strain SHPJ‐2

Combined with the binning analysis of consortium PDMC, we speculated that bin9, annotated as the order *Sphingomonadales*, with 100% completeness and abundant xenobiotics metabolic genes, was most likely to be the reference metagenomic assembled genome (MAG) for strain SHPJ‐2. Generally, sphingomonads contain abundant terminal oxygenases and are well‐known for their ability to degrade many aromatic compounds^7^, ^8^. A similar gene‐encoding phenomenon was also found in strain SHPJ‐2. A total of seven sets of putative α‐ and β‐subunit RHD genes (*orf5103–orf5104, orf3136–orf3137, orf3864–orf3865, orf3869–orf3870, orf3872–orf3873, orf3890–orf3891*, and *orf4249–orf4250*), eight putative genes coding for P450s (*orf1725, orf4073, orf5095, orf5178, orf3192, orf3194, orf3199*, and *orf3927*), two ferredoxin‐encoding genes (*orf4248* and *orf5105*) and two ferredoxin reductase‐encoding genes (*orf4252* and *orf5102*) were identified by Rapid Annotation Subsystem Technology (RAST) annotation and Basic Local Alignment Search Tool (BLAST) analysis.

**Figure 4:**
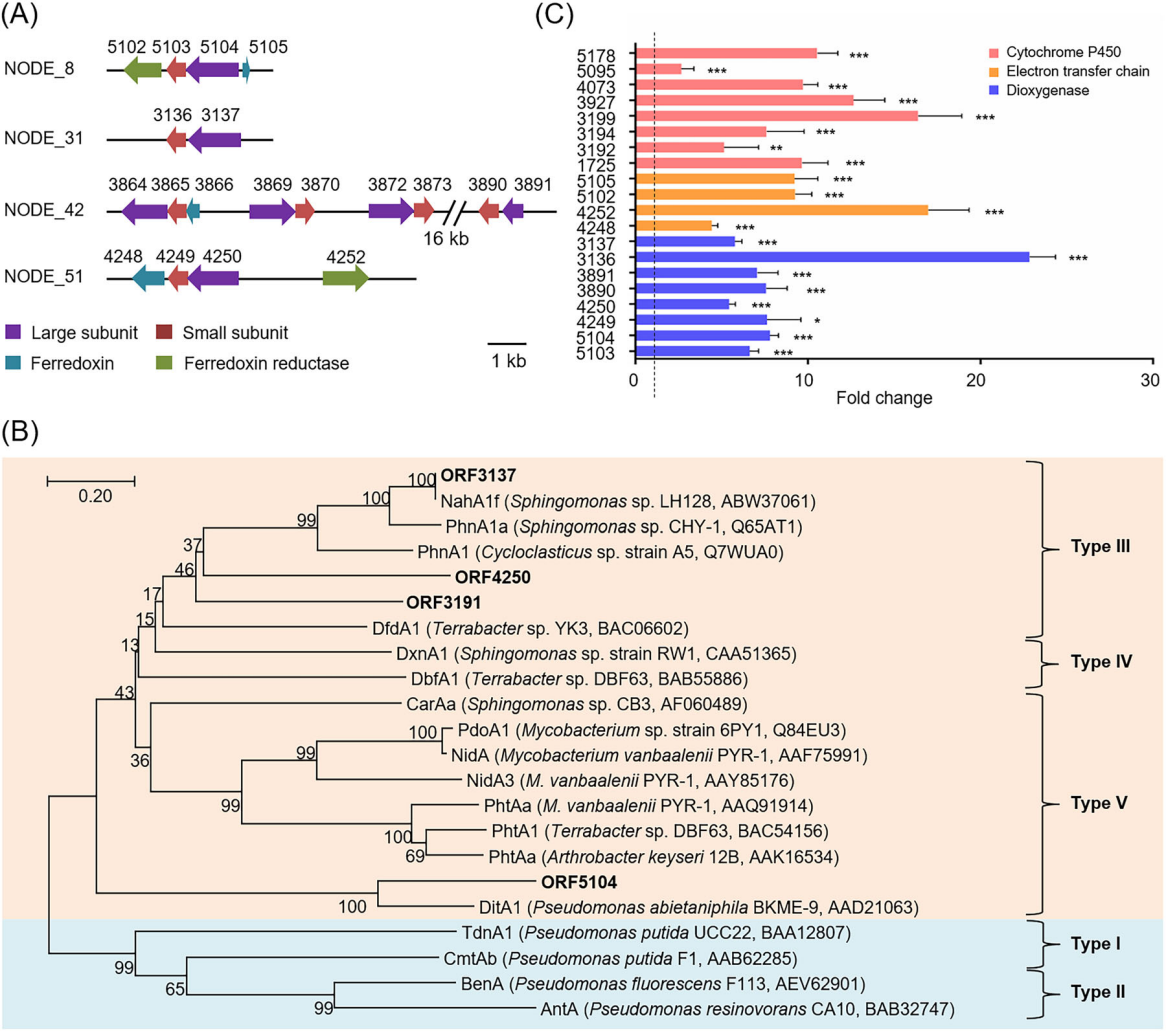
Characterization of genes associated with PHE degradation from strain SHPJ‐2. (A) Structural diagram of the predicted genes coding RHDs in strain SHPJ‐2. Purple, red, blue, and green represent the α‐subunit, β‐subunit, ferredoxin, and ferredoxin reductase, respectively. (B) Phylogenetic analysis of predicted RHDs in strain SHPJ‐2 with other characterized RHDs. RHD types were classified^37^ and different functions are indicated by different colors. Orange and blue represent the RHDs responsible for catalyzing PAHs and heterocyclic derivatives, benzoates, and their substituted benzoates, respectively. (C) Relative expression levels of transcriptional changes of genes related to PHE degradation. Red, yellow, and blue represent the coding genes of P450s, electron transfer chain components, and RHDs, respectively; significance analysis is one‐way analysis of variance; dotted line shows one‐fold relative expression levels. PAH, polycyclic aromatic hydrocarbon; RHD, ring‐hydroxylating dioxygenase. *p < 0.05; **p < 0.01; ***p < 0.001.

The organization of the genes encoding RHDs and the electron transfer chain is displayed in Figure 4A. Genes encoding RHDs, except for *orf3864–orf3865*, were arranged in a similar genetic fashion, that is, the α‐subunit precedes the β‐subunit. Of them, the products of *orf3137* and *orf4250* displayed relatively high similarities (>90%) with the α‐subunit of RHDs in *Sphingomonas* sp. LH128^19^ and *Novosphingobium guangzhouense* ^48^, respectively. The α‐subunits of RHDs encoded by *orf5104* and *orf3891* were highly conserved in the family *Sphingomonadaceae* with at least 79% amino acid similarities. Three pairs of RHDs, ORF3864–ORF3865, ORF3869–ORF3870, and ORF3872–ORF3873, shared 100% amino acid similarity with the salicylate‐1‐hydroxylases AdhA1cA2c, XylXY, and AdhA1dA2d, respectively, in the *Sphingobium* sp. PNB strain^49^. Therefore, a phylogenetic tree was constructed using the remaining four α‐subunits of RHDs (ORF3137, ORF4250, ORF5104, and ORF3891) and other characterized RHDs (Figure 4B). All four α‐subunits of RHDs in strain SHPJ‐2 are clustered with the RHDs catalyzing PAHs and heterocyclic derivatives. Among them, ORF4250, ORF3137, and ORF3191 are more closely related to NahA1f (*Sphingomonas* sp. LH128, ABW37061)^19^, PhnA1a (*Sphingomonas* sp. CHY‐1, Q65AT1)^18^, and PhnA1 (*Cycloclasticus* sp. strain A5, Q7WUA0)^50^, all of which belong to type III RHDs^38^. In addition, ORF5104 is in the same branch as DitA1 (AAD21063)^51^ which belongs to type V RHDs consisting of a [3Fe‐4S]‐type ferredoxin and a GR‐type reductase. Therefore, it can be speculated that ORF4250, ORF3137, and ORF3191 belong to type III RHDs, while ORF5104 belongs to type V RHDs. We performed further functional confirmation for the four pairs of RHD genes: *orf3136‐orf3137*, *orf4249‐orf4250*, *orf5103‐orf5104*, and *orf3890‐orf3891*.

Similarly, genes *orf5105* and *orf4252* were thought to encode ferredoxin due to their high levels of amino acid homology to their homologs (>73%) in several strains from the family *Sphingomonadaceae*. In addition, the predicted gene *orf5102* was found to encode a FAD‐dependent oxidoreductase; the *orf4248* product was annotated as the α‐subunit of dioxygenase, but its amino acid sequence shared 97% homology with the Rieske [2Fe‐2S] domain‐containing protein of *Sphingobium* sp. C100 (Figure S2)^52^. Altogether, we speculated that the proteins expressed by *orf5102‐orf5105* and *orf4248‐orf4252* could be functional components of the electron transfer chain, which requires further study.

Meanwhile, a phylogenetic analysis of the eight predicted P450s with the reported PAHs‐degrading P450s revealed that the individual P450s in strain SHPJ‐2 were distantly related (Figure S3). The homology of these P450s with those in Uniprot ranged from 83% to 99%, in which the lowest amino acid homology was between ORF3927 and its similar proteins in *Sphingobium baderi* (A0A0S3F3E3). In contrast, the highest amino acid similarity was between ORF5178 and its homologs in *Sphingomonas* sp. strain SKA58 (Q1NHG2).

To identify the above‐mentioned potential genes associated with the PAH degradation, reverse‐transcription quantitative polymerase chain reaction (RT‐qPCR) assays comparing the control group (with glycerol as the sole carbon source) and the treatment group (with PHE as the sole carbon source) were constructed. As shown in Figure 4C, four pairs of RHDs genes (*orf5103‐orf5104, orf3136‐orf3137, orf3890‐orf3891*, and *orf4249‐orf4250*), two sets of genes for electron transfer chain components (*orf5102‐orf5105* and *orf4248‐orf4252*), and eight P450s genes (*orf1725, orf4073, orf5095, orf5178, orf3192, orf3194, orf3199* and *orf3927*) were significantly upregulated in the presence of PHE. These results indicate that these genes were directly associated with the PHE degradation process by *Sphingobium* sp. SHPJ‐2.

### Functional verification of possible RHDs and P450s of strain SHPJ‐2

To confirm the specific role of the above‐mentioned genes during PHE degradation, we amplified and cloned each gene into the appropriate plasmid. Specifically, genes encoding RHDs or P450s were ligated into the plasmid pET‐28a(+), while genes encoding ferredoxin and ferredoxin reductase were cloned into a pET‐28a(+)‐compatible plasmid, pACYCDuet‐1. The constructed plasmids were validated by PCR with universal primers (Figure S4). Furthermore, a sodium dodecyl‐sulfate polyacrylamide gel electrophoresis (SDS‐PAGE) analysis of the recombinant plasmids demonstrated that all proteins were significantly expressed in *Escherichia coli* BL21(DE3) and were consistent with the predicted molecular masses (Figure S5).

**Figure 5:**
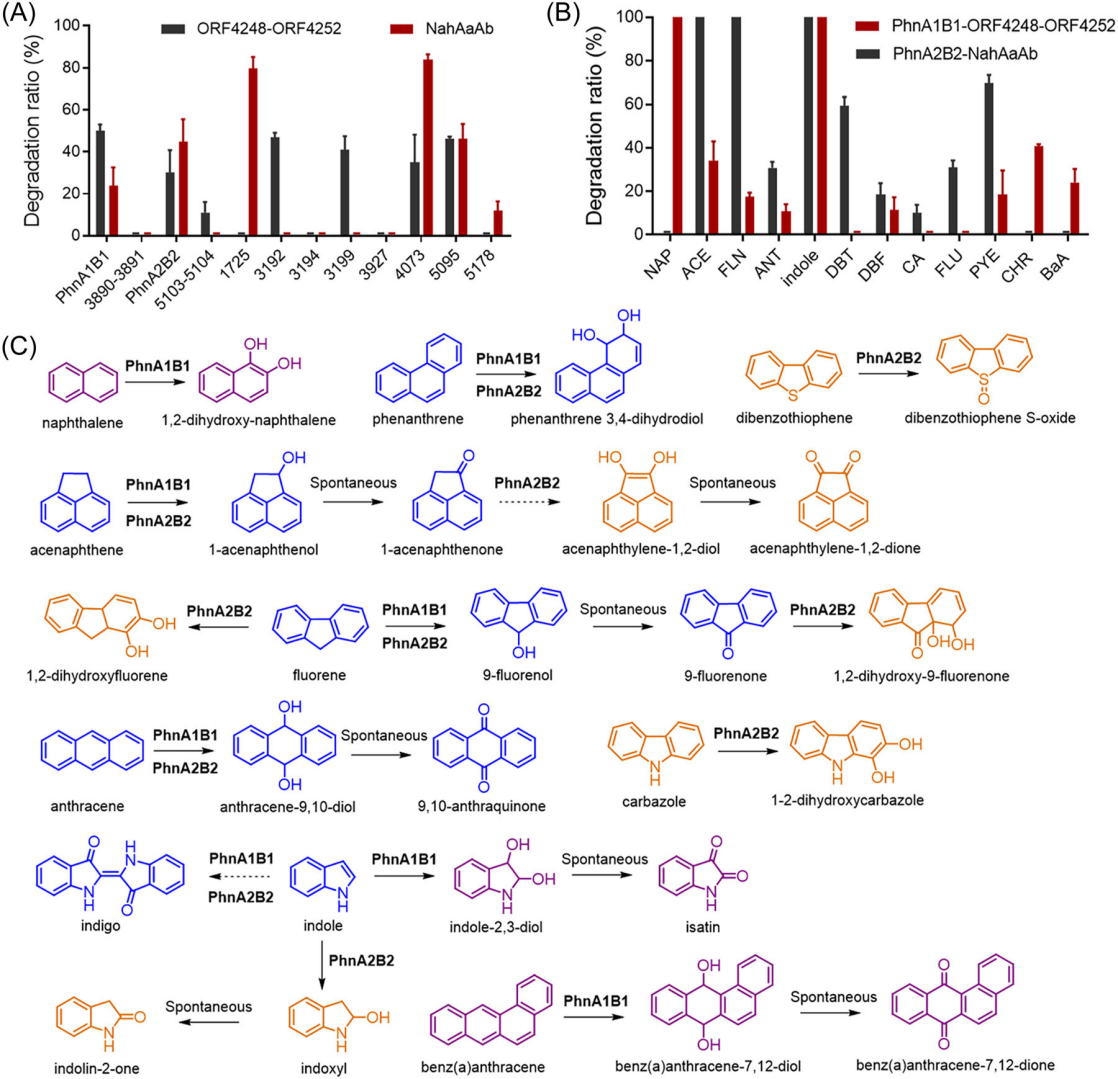
Degradation characteristics of PhnA1B1 and PhnA2B2 from strain SHPJ‐2. (A) Degradation of PHE at 24 h by oxygenase recombinant strains expressing electron transfer chain ORF4248–ORF4252 or NahAaAb. (B) The catalytic efficiency of PhnA1B1 co‐expressed with ORF4248‐ORF4252 and PhnA2B2 co‐expressed with NahAaAb on PAHs at 24 h. (C) Proposed metabolic pathways of PAHs with known product structures catalyzed by PhnA1B1 or PhnA2B2. The proposed pathways of FLU, PYE, and DBF are not shown in this figure due to uncertainty in their product structures. The blue compounds were produced by both RHDs, while the purple and orange compounds were only catalyzed by PhnA1B1 and PhnA2B2, respectively. The solid and dotted arrows represent one‐step or multistep reactions, respectively. ORF, open reading frame.

The function of these genes was verified *in vivo* by the resting cell reactions. As shown in Table S3, the PHE concentration was essentially unchanged in recombinant strains expressing only oxygenase but lacking electron transfer chains, indicating that the endogenous electron transport chain from *E. coli* cannot provide appropriate reducing power. This phenomenon was consistent with the reported expression of several RHDs in *E. coli*, such as NidAB^17^, NidA3B3^17^, PhnA1aA2a^18^, and PhnA1fA2f^19^. Although ORF4248–ORF4252, ORF5102–ORF5105, and NahAaAb were all type III electron transport chains consisting of a [2Fe‐2S]‐type ferredoxin and an FNR_N_‐type reductase^38^, ORF5102–ORF5105 did not provide adequate reducing power for any of the target genes. This is likely because ORF5105 (7.1 kD) is much smaller than the other reported reductases (~30 kD) and, therefore, lacks the relevant functional domain, leading to malfunction. However, the recombinant strains with the electron transfer chain ORF4248–ORF4252 or NahAaAb exhibited different PHE degradation efficiencies (Figure 5A). Specifically, the oxygenases ORF3890–ORF3891, ORF3194, and ORF3927 showed no degradation activity regardless of which electron transfer chain they were co‐expressed with. The oxygenases ORF5103–ORF5104, ORF3192, and ORF3199 degraded PHE only when they were co‐expressed with ORF4248–ORF4252, while ORF1725 and ORF5178 functioned only in the presence of NahAaAb. This suggests that even the electron transfer chain components from the same genus may not provide optimal reducing power for the RHDs of the strain. ORF3136–ORF3137, ORF4249–ORF4250, ORF4073, and ORF5095 showed different PHE degradation efficiencies when combined with different electron transfer chains, demonstrating that the appropriate electron transfer chain components were essential for the proper function of the oxygenases^17^. In addition, two type V electron transfer chains, PhtAcAd from *Mycobacterium vanbaalenii* PYR‐1 and phdCD from *Nocardioides* sp. KP7^17^, were largely incompatible with the oxygenases in strain SHPJ‐2.

The PHE products were accumulated using recombinant bacteria with the optimal degradation ratio. Samples showing a distinct bright yellow color at 24 h were extracted, concentrated, derived with *N*,*O*‐bis(trimethylsilyl)trifluoroacetamide (BSTFA), and detected by GC‐MS. As shown in Table 2, three monohydroxy metabolites (17.485, 17.554, and 17.774 min), *cis*‐3,4‐dihydrodiol (18.432 min), and another dihydrodiol metabolite (19.703 min) were detected in the ORF4249–ORF4250 reaction system based on previous studies^53^. Nevertheless, the structures of the above‐mentioned monohydroxy and dihydrodiol metabolites could not be determined due to the lack of available information. This suggests that the ORF4249–ORF4250 transformed PHE through at least two neighboring positions, one of which was at the C‐3,4 position. In addition, another RHD, ORF3136–ORF3137, could transform PHE into *cis*‐3,4‐dihydrodiol metabolite, a common product of PHE degradation catalyzed by bacterial RHDs^37^. All remaining P450s, including ORF1725, ORF3192, ORF3199, ORF4073, and ORF5095, converted PHE to *cis*‐3,4‐dihydrodiol (18.432 min) in the recombinant bacteria. In general, P450s act as monooxygenases and catalyze the production of PAHs monohydroxy metabolites^13^, ^14^. Unusually, the catalytic products detected from the above‐mentioned five P450s were all dihydroxylated products. Not coincidentally, Bubinas et al. found a similar phenomenon in the NAP metabolite assays: P450s first catalyzed the production of 1‐naphthol and 2‐naphthol, followed by a second catalytic step to generate 1,2‐dihydroxynaphthalene^54^. Therefore, we hypothesized that P450s expressed by *orf1725, orf3192, orf3199, orf4073*, and *orf5095* could also catalyze similar reactions, such as transforming PHE to monohydroxy products (3‐hydroxy PHE and 4‐hydroxy PHE) and continuing the monooxygenation reaction to produce PHE‐3,4‐dihydrodiol. Unfortunately, we did not detect any PHE products by GC‐MS in ORF5103–ORF5104 or ORF5178 recombinant strains, likely due to their low PHE transformation efficiency and the lower product content.

**Table 2:** GC‐MS results of PHE metabolites produced by predicted oxygenases.

ORF	Proposed product	Retention time (min)	Mass spectral characteristics of product (ion abundances)
4249–4250	Monohydroxy‐PHE (TMS)	17.485	266 (999), 251 (641), 184 (226), 73 (186), 165 (184), 252 (166), 85 (149)
4249–4250	Monohydroxy‐PHE (TMS)	17.554	266 (999), 251 (711), 235 (361), 267 (243), 165 (179), 73 (171), 252 (153), 176 (106)
4249–4250	Monohydroxy‐PHE (TMS)	17.774	266 (999), 251 (826), 267 (237), 165 (190), 252 (180), 176 (167), 57 (125), 73 (118)
4249–4250	PHE‐3,4‐dihydrodiol (2TMS)	18.432	73 (999), 354 (973), 266 (350), 355 (302), 236 (203), 356 (113), 57 (78), 267 (77)
4249–4250	PHE dihydrodiol (2TMS)	19.703	73 (999), 354 (995), 266 (277), 236 (230), 71 (155), 85 (135), 355 (123)
3136–3137, 1725, 3192, 3199, 4073 and 5095	PHE‐3,4‐dihydrodiol (2TMS)	18.432	73 (999), 354 (973), 266 (350), 355 (302), 236 (203), 356 (113), 57 (78), 267 (77)

### PhnA1B1 and PhnA2B2 have different substrate specificities

We named two RHDs with better PHE degradation efficiency, ORF3136–ORF3137 and ORF4249–ORF4250, as PhnA1B1 and PhnA2B2, respectively. The substrate specificities of both RHDs were investigated by testing 12 aromatic substrates separately. As shown in Figure 5B, when PhnA1B1 was co‐expressed with ORF4248–ORF4252, its resting cell system could catalyze the degradation of nine substrates: NAP, ACE, FLN, ANT, indole, DBF, PYE, CHR, and BaA. Of them, NAP and indole were completely degraded within 24 h, and the degradation ratios of the other PAHs ranged from 15.1% to 45.3%. PhnA2B2 degraded ACE, FLN, ANT, indole, DBT, DBF, CA, FLU, and PYE, in the presence of NahAaAb. Of these, ACE, FLN, and indole were completely degraded, while the catalytic efficiencies of PYE and DBT were approximately 70% and 60%, respectively. The degradation efficiencies of the remaining substrates were approximately 30%.

The properties of PAH metabolites catalyzed by PhnA1B1 are displayed in Table 3. 1‐Naphthalenol and 2‐naphthalenol were observed in the NAP samples, suggesting that the major isomer formed was *cis*‐1,2‐dihydrodiol. In the ACE samples, we detected only one product, 1‐acenaphthenone, which should be generated by the oxidation of 1‐acenaphthenol by endogenous dehydrogenase. In the case of FLN, 9‐fluorenol and another monohydroxy product were detected, eluting at 14.176 and 14.748 min, respectively. In addition, 9,10‐anthraquinone was detected in the ANT sample, and BaA‐7,12‐dione (21.366 min) and *cis*‐7,12‐dihydrodiol (22.712 min) were generated when BaA was used as the substrate. A distinct blue color was observed in the medium when indole was added, while a peak with a retention time of 13.748 min was detected as the isatin. This indicates that the 2,3‐dihydroxyindole was generated but immediately transformed to isatin by endogenous dehydrogenase. We did not detect the transformation products of CHR, PYE, and DBF catalyzed by PhnA1B1.

**Table 3:** GC‐MS results of PAH metabolites produced by PhnA1B1.

Substrate	Proposed product	Structure	Retention time (min)	Mass spectral characteristics of product (ion abundances)
NAP	1‐Naphthalenol	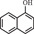	11.849	115 (999), 144 (967), 116 (480), 89 (138), 63 (132), 145 (108), 39 (74)
	1‐Naphthalenol (TMS)		12.184	216 (999), 201 (993), 185 (595), 73 (444), 115 (318), 141 (219), 127 (218)
	2‐Naphthalenol	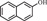	11.935	144 (999), 115 (706), 116 (240), 63 (109), 145 (103), 89 (107), 39 (62), 62 (56)
	2‐Naphthalenol (TMS)		12.403	201 (999), 216 (705), 73 (188), 145 (188), 202 (177), 127 (170), 115 (140)
ACE	1‐Acenaphthenone	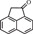	13.541	168 (999), 140 (858), 139 (757), 69 (155), 169 (139), 63 (127), 70 (102), 141 (101)
FLN	9‐Fluorenol	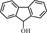	14.176	181 (999), 182 (777), 152 (548), 151 (197), 153 (196), 165 (177), 76 (141), 183 (99)
	Hydroxy‐FLN (TMS)	—	14.748	165 (999), 254 (412), 253 (206), 163 (148), 73 (147), 239 (101), 139 (54)
ANT	9,10‐Anthraquinone	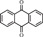	16.284	208 (999), 180 (994), 152 (896), 151 (475), 76 (355), 50 (205), 74 (177)
BaA	Benz(a)anthracene‐7,12‐dione	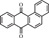	21.366	258 (999), 101 (551), 202 (484), 100 (402), 230 (362), 200 (317), 88 (273)
	Benz(a)anthracene‐7,12‐dihydrodiol (2TMS)	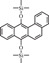	22.712	404 (999), 73 (378), 45 (53), 315 (45), 285 (37), 287 (36), 389 (35)
Indole	Isatin	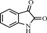	13.748	119 (999), 92 (634), 147 (433), 64 (233), 63 (172), 91 (162), 57 (133), 62 (120)

The specific metabolite profiles of PhnA2B2 related to PAH degradation are listed in Table 4. PhnA2B2 converted FLN to 9‐fluorenol (14.748 min in derived sample), 9‐fluorenone (14.222 min), 2‐fluorenol (15.868 min), and 2‐hydroxy‐9‐fluorenone (17.289 min), indicating that it can transform FLN through the C‐1,2 and C‐9 positions. Four ACE metabolites were found at 13.500 min (1‐acenaphthenol), 13.465 min (1‐acenaphthenone), 15.423 min (acenaphthylene‐1,2‐dione), and 16.619 min (*cis*‐1,2‐dihydrodiol). Therefore, we speculated that PhnA2B2 could successively generate monohydroxy and dihydroxy ACE by sequential reactions, while 1‐acenaphthenone and acenaphthylene‐1,2‐dione were most likely to be generated by the conversion of hydroxylated substances under *E. coli* endogenous dehydrogenase^37^. The metabolite profile for the degradation of ANT was identical to that of PhnA1B1. Furthermore, PhnA2B2 could transform DBT, CA, and indole into the corresponding products, namely, monohydroxy‐DBT (17.583 min) and dibenzothiphene‐*S*‐oxide (17.341 min); 2‐hydroxycarbazole (17.751 min); indigo, indoxyl (12.114 min), and indolin‐2‐one (12.033 min); respectively. Several monohydroxy metabolites were identified as spontaneous dehydration products of the dihydrodiol products in each of the FLU, PYE, and DBF conversions, though their specific structures were difficult to determine.

**Table 4:** GC‐MS results of PAH metabolites produced by PhnA2B2.

Substrate	Proposed product	Structure	Retention time (min)	Mass spectral characteristics of product (ion abundance)
ACE	1‐Acenaphthenol	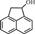	13.500	169 (999), 170 (874), 141 (434), 152 (386),115 (349), 153 (315), 139 (274)
	1‐Acenaphthenone	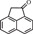	13.465	168 (999), 140 (858), 139 (757), 69 (155), 169 (139), 63 (127), 70 (102), 141 (101)
	Acenaphthylene‐1,2‐dione	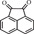	15.423	126 (999), 154 (936), 182 (529), 74 (136), 127 (126), 63 (134), 155 (114)
	ACE‐1,2‐dihydrodiol (2TMS)	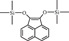	16.619	328 (999), 73 (620), 329 (302), 240 (134), 45 (123), 330 (106), 210 (81)
FLN	9‐Fluoreone	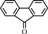	14.222	180 (999), 152 (466), 151 (218), 76 (204), 181 (138), 150 (131), 63 (122), 126 (79)
	2‐Fluorenol	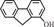	15.868	182 (999), 181 (584), 152 (385), 153 (179), 165 (135), 76 (74), 126 (47)
	2‐Hydroxy‐9‐fluorenone	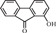	17.289	196 (999), 139 (499), 197 (146), 140 (130), 168 (92), 113 (62), 84 (54), 63 (48)
	9‐Fluorenol (TMS)	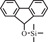	14.748	165 (999), 254 (412), 253 (206), 163 (148), 73 (147), 239 (101), 139 (54)
	2‐Fluoreonl (TMS)	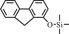	16.226	254 (999), 239 (700), 165 (281), 240 (162), 152 (146), 73 (119), 163 (99), 178 (99)
ANT	9,10‐Anthraquinone	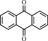	16.284	208 (999), 180 (994), 152 (896), 151(475), 76(355), 50(205), 74 (177)
	ANT dihydrodiol	—	17.451	165 (999), 166 (968), 212 (384), 194 (357), 152 (253), 140 (230), 181 (219)
	Monohydroxy‐ANT (TMS)	—	18.843	354 (999), 73 (488), 355 (322), 356 (120), 75 (103), 353 (99), 266 (80), 235 (72), 117 (68)
FLU	Monohydroxy‐FLU (TMS)	—	20.084	290 (999), 275 (543), 73 (256), 291 (346), 189 (242), 57 (175), 200 (161)
PYE	Monohydroxy‐PYE (TMS)	—	20.361	290 (999), 73 (301), 291 (259), 275 (250), 189 (231), 274 (153), 187 (106), 57 (104)
DBF	Monohydroxy‐DBF (TMS)	—	15.227	184 (999), 128 (238), 127 (158), 185 (137), 155 (117), 102 (98), 126 (80), 74 (53)
		—	15.308	184 (999), 128 (238), 57 (218), 127 (204), 55 (181), 155 (149), 185 (127)
DBT	Monohydroxy‐DBT (TMS)	—	17.583	171 (999), 200 (860), 172 (584), 184 (290), 139 (217), 168 (132), 201 (127), 127 (105)
	Dibenzothiophene‐*S*‐oxide	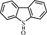	17.341	184 (999), 139 (182), 152 (101), 200 (92), 57 (56), 69 (78), 92 (66)
CA	2‐Hydroxycarbazole	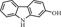	17.751	183 (999), 154 (333), 182 (149), 184 (137), 127 (127), 128 (93), 77 (69)
	2‐Hydroxycarbazole (TMS)	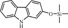	17.942	255 (999), 240 (546), 256 (212), 73 (154), 241 (121), 180 (114), 212 (109)
Indole	Indoxyl (TMS)	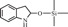	12.114	205 (999), 73 (714), 190 (666), 206 (184), 162 (173), 132 (134), 77 (113), 45 (107)
	Indoxyl (2TMS)	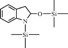	14.517	277 (999), 73 (672), 189 (235), 45 (119), 146 (82), 262 (64)
	Indolin‐2‐one	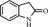	12.033	104 (999), 133 (975), 73 (467), 105 (420), 51 (193), 77 (183), 63 (76)

The degradation properties and substrate specificities of PhnA1B1 and PhnA2B2 can be summarized as follows: first, PhnA1B1 and PhnA2B2 had different substrate preferences. Both RHDs catalyze a wide range of substrates, including PHE, ACE, FLN, ANT, PYE, DBF, and indole. PhnA2B2 can also degrade CA, DBT, and FLU, while PhnA1B1 is capable of degrading NAP, CHR, and BaA (Figure 5A,B). Despite limited oxidation activities, it is worth noting that both enzymes showed differentially preferred catalytic capacities for the four‐ring PAHs, such as PYE, FLU, CHR, and BaA. PhnA1 of strain SHPJ‐2 shared 99% amino acid homology with PhnA1f, the α‐subunit of RHD in *Sphingomonas* sp. LH128, differing only at residue 198. PhnA1fA2f co‐expressed with PhnA3A4, the electron transfer chain component from *Sphingomonas* sp. CHY‐1, can oxidize NAP, PHE, FLN, ANT, FLU, BaA, PYE, CHR, DBT, and DBF, but not ACE, benzo[*k*]fluoranthene, and benzo[*e*]fluoranthene^19^. Analysis of PhnA2B2 and PhnA1fA2f revealed that the substrate specificities of the two enzymes largely overlapped, except for ACE and FLU. PhnA1fA2f can catalyze FLU but not ACE, while PhnA2B2 did the opposite. The difference in the substrate profiles of these two RHDs indicates that the subtle difference in residue 198 (substitution of threonine for alanine) can affect the structure of the catalytic pocket of PhnA2 and thus the substrate specificities. Similarly, PhnA2 of strain SHPJ‐2 displayed relatively high similarities (>90%) with the α‐subunit of RHDs of *Novosphingobium guangzhouense*, which reportedly degrades 1‐methyl PHE^48^. Unfortunately, no studies related to the molecular mechanism of *Novosphingobium guangzhouense* were found.

Second, PhnA1B1 and PhnA2B2 can catalyze monooxygenation or sulfoxidation. For example, both RHDs oxidized ACE and FLN to 1‐acenaphthenol and 9‐fluorenone, respectively, via monohydroxylation, and PhnA2B2 also catalyzed the monohydroxylation of indole to indoxyl and the sulfoxidation of DBT to dibenzothiophene‐*S*‐oxide. Furthermore, PhnA1B1 can regiospecifically oxidize all substrates (Table 3). On the contrary, PhnA2B2 catalyzed PHE and FLN at relatively low regiospecificities, producing several isomeric dihydrodiols or monohydroxy metabolites, while the other substrates were regiospecifically oxidized and produced only one kind of metabolite (Table 4).

Proposed metabolic pathways of PAHs with known product structures catalyzed by PhnA1B1 or PhnA2B2 are shown in Figure 5C. Of note, some products that were not detected in the resting cell reaction of strain SHPJ‐2, including PHE‐3,4‐dihydrodiol, 1,2‐dihydroxyfluorene, 9‐fluorenol, 1,2‐dihydroxycarbazole, and benzo(a)anthracene‐7,12‐dihydrodiol, were detected in the catalytic system of RHDs. These results enriched the corresponding intermediate metabolites and provided strong evidence for the metabolic pathways of strain SHPJ‐2.

In summary, we first analyzed the metagenomic binning of bacterial consortium PDMC and obtained a *Sphingomonadales* assembly genome with 100% completeness. Meanwhile, the strain SHPJ‐2 with multiple PAH degradation capabilities was successfully isolated and characterized from bacterial consortium PDMC. Based on the prediction of order and xenobiotics metabolic genes, the *Sphingomonadales* assembly genome was most likely to be the MAG for strain SHPJ‐2. On this basis, several important RHDs or P450s responsible for the PAH degradation were identified. Among them, PhnA1B1 and PhnA2B2 are two important RHDs with different PAH catalytic efficiencies and substrate preferences and could degrade some HMW‐PAHs such as PYE, FLU, CHR, and BaA. These results provides detailed insights into how strain SHPJ‐2 degraded various PAHs and lay an important foundation for subsequent genetic utilization and bioremediation.

## MATERIALS AND METHODS

### Chemicals and media

PAHs and heterocyclic compounds (PHE, NAP, ACE, FLN, ANT, FLU, BaA, PYE, CHR, DBT, DBF, CA, and indole) used for the degradation assay were purchased from Aladdin or J&K Scientific. Standards of metabolic intermediates, such as 1‐hydroxy‐2‐naphthoic acid, salicylic acid, 9‐fluorenol, 9‐fluorenone, indigo, and dibenzothiophene‐*S*‐oxide, were purchased from either Macklin or Sinopharm Chemical Reagent Co., Ltd. All water‐insoluble substrates were dissolved in *N*,*N*‐dimethylformamide (DMF) as stock solutions and the appropriate volume was added to the medium before use. BSTFA for derivatization of samples was purchased from Sigma‐Aldrich. The purity of all chemicals exceeded 99%. The PHE degraders were cultured with an MSM with PHE as the sole carbon source. MSM per liter consists of 3.7 g KH_2_PO_4_, 5.2 g K_2_HPO_4_·3H_2_O, 2.0 g NH_4_Cl, 1.0 g Na_2_SO_4_, 0.1 g MgSO_4_, and 1 ml trace metal solution^44^. Luria–Bertani (LB) broth medium (tryptone 1%, yeast extract 0.5%, and NaCl 1%) was used for the cultivation of *E. coli* and was supplemented with antibiotics when necessary. All abbreviations in the text are listed in Table S4.

### Metagenomic DNA extraction and shotgun sequencing of consortium PDMC

The bacterial consortium PDMC enriched from the PAHs‐contaminated environment was capable of growing with PHE as the sole carbon source and degrading several PAHs and heterocyclic compounds^21^. The consortium PDMC was collected in the late logarithmic stage (48 h) and cultured in MSM containing 400 mg/L PHE. Total genomic DNA of consortium PDMC was extracted using a DNeasy PowerSoil Kit (Qiagen Inc.). The quantity and quality of the extracted DNA were assayed by NanoDrop ND‐1000 spectrophotometer (Thermo Fisher Scientific) and agarose gel electrophoresis, respectively. The metagenome shotgun sequencing libraries with insert sizes of 400 bp were constructed with an Illumina TruSeq Nano DNA LT Library Preparation Kit. Each library was sequenced by the Illumina NovaSeq platform (Illumina) using the PE150 strategy at Personal Biotechnology Co., Ltd.

### Sequence assembly and scaffold binning

A valid sequence set used for subsequent assembly was obtained after trimming and filtering the raw sequencing data by Cutadapt ver 1.2.1^55^. Assembly was performed using IDBA‐UD^56^ with a minimum contig size of 300 bp. The genes and ORF of assembled scaffolds were predicted using MetaGeneMark^57^. Meanwhile, binning analysis was performed on the assembled scaffolds using MetaWRAP^58^. The integrity and pollution degree of each resulting genome bin was evaluated by CheckM^59^; with the integrity and pollution rate parameters set at 80 and 10, respectively. The Classify_Bins module in MetaWRAP was used to identify the species of each genome bin. Prediction and annotation of functional genes were performed using the RAST (http://rast.nmpdr.org/org/)^60^, KAAS (https://www.genome.jp/tools/kaas/)^61^, and BLAST (https://www.ncbi.nlm.nih.gov/BLAST)^62^.

### Isolation and identification of PHE degraders from consortium PDMC

The strains from consortium PDMC were isolated on the MSM plates with PHE as the sole carbon source using the serial dilution spread plate method. Repeating the incubation for several times, two colonies in white and yellow color were isolated from the MSM plates, termed as SHPJ‐1 and SHPJ‐2, respectively. All cultivations were performed in MSM containing 400 mg/L PHE as the sole carbon source at 30°C, with shaking at 200 rpm. The species of strains were identified by 16S rRNA sequences, which were amplified using universal primers 27F and 1492R. The phylogenetic tree was constructed by MEGA X^63^ using the neighbor‐joining method with bootstrap analysis of 1000 replicates.

### Characterization of PHE degradation of strain SHPJ‐2

Both PHE degraders (SHPJ‐1 and SHPJ‐2) were grown in MSM containing 400 mg/L PHE. By detecting the final PHE concentration after 3 days of cultivation, strain SHPJ‐2 was chosen for in‐depth study due to its higher PHE degrading efficiency. To study the influences of different parameters on the degradation performance of strain SHPJ‐2, 5% (v/v) seed broth was inoculated into MSM containing 400 mg/L PHE at pH 7 and incubated at 30°C and 200 rpm. The seed broth was subsequently transferred and one of the incubation conditions was altered to compare degradation ratios, such as PHE initial concentration, pH values, inoculum size, and temperature. The PHE content was detected on the 3rd day to determine the optimal degradation conditions for the SHPJ‐2 strain. The growth and PHE degradation were also detected under optimal conditions using previously reported methods^21^.

To study the expression of PAH degradation‐related genes in strain SHPJ‐2, bacteria were cultured with MSM containing 200 mg/L PHE or 1% glycerol (v/v) and collected at the logarithmic phase. The resting cells were prepared according to previously outlined methods^21^. We then added 400 mg/L PHE to each reaction system and recorded the PHE content over time.

### Characterization of PAH and heterocyclic derivative degradation

When the SHPJ‐2 strain was cultured with MSM containing PHE as the sole carbon source, its cells were collected in the late logarithmic phase and prepared as the resting cells. Different substrates (PAHs or heterocyclic derivatives) were added to 20 ml of the reaction systems for the degradation experiments. The final concentrations of ACE, NAP, DBF, DBT, FLN, CA, and indole were all 100 mg/l, and the final concentrations of PYE, FLU, CHR, and BaA were 5 mg/l, and that of ANT was 20 mg/l. A blank control containing only the substrate and an inactive cell control using autoclaved incubations with substrates were set up in each group. All reactions were incubated at pH 7°C and 30°C. The residual concentrations of substrates at different reaction time (0, 24, and 48 h) were extracted with an equal volume of ethyl acetate and measured by high‐performance liquid chromatography (HPLC; Agilent Technologies 1200 series). The HPLC was performed by the Agilent Eclipse XDB‐C18 column (4.6 × 150 nm; 5 μM), using previously described methods^21^. At least three experimental replicates were implemented for each degradation test.

### Determination of intermediate metabolites

Samples with reaction time of 0, 24, and 48 h were taken for intermediate metabolite detection. The intermediate metabolites from various degradation samples (neutral and acidic samples) were extracted by ethyl acetate, dehydrated by anhydrous Na_2_SO_4_, concentrated with a rotary evaporator, and dried with high‐purity N_2_. The dried samples were dissolved in 500 μl of chromatographic‐grade DMF. The re‐dissolved samples were detected by GC‐MS (Agilent & GC‐7890B; MS‐5977B), while the BSTFA‐derived redissolved samples were detected in the same way. A GC‐MS system equipped with an HP‐5MS column (30 m × 0.25 mm, 0.25 μM) was used to detect the intermediate metabolites of PAHs with the following oven heating program: maintained at 75°C for 3 min, increased to 250°C at a rate of 12°C/min and maintained for 1 min, then increased to 300°C at a rate of 10°C/min and maintained for 10 min. The indigo sample was detected using the previous method^64^.

### RT‐qPCR analysis

Strain SHPJ‐2 was induced to degrade PHE when PHE was the sole carbon source in MSM; therefore, the strain can be inoculated into MSM containing 1% (v/v) glycerol or 400 mg/l PHE as the control and treatment groups, respectively, to determine the changes in the transcript levels of the relevant degradation genes. The bacteria were cultured at 30°C and collected during the mid‐to‐late logarithmic period. Total RNA was extracted with TIANGEN RNAprep Pure (TianGen). Reverse transcription was performed using a Hifair® II 1st Strand cDNA synthesis Kit (Yeason) after the extracted RNA was treated with DNase I (Thermo Fisher Scientific). RT‐qPCR was conducted with a CFX96 Real‐time PCR Detection System (Bio‐Rad) with Hifair® qPCR SYBR® Green Master Mix (No Rox) (Yeason). The RT‐qPCR primers are listed in Table S5. The relative expression level of each gene was calculated according to the following formula: $\mathrm{ratio}=\frac{{E_{\mathrm{target}}}^{∆C_{\mathrm{Ttarget}}(\mathrm{control}-\mathrm{sample})}}{{E_{\mathrm{ref}}}^{∆C_{\mathrm{Tref}}(\mathrm{control}-\mathrm{sample})}},$ where 16S rRNA was used as the reference gene. Statistical analysis was performed using one‐way analysis of variance.

### Construction of plasmids for functional analysis

The four presumed genes encoding RHDs (*orf3136‐3137*, *orf3890‐3891*, *orf4249‐4250*, and *orf5103‐5104*), eight putative P450s genes (*orf1725*, *orf3192*, *orf3194*, *orf3199*, *orf3927*, *orf4073*, *orf5095*, and *orf5178*), two ferredoxin‐encoding genes (*orf4248* and *orf5105*) and two ferredoxin reductase‐encoding genes (*orf4252* and *orf5102*) were amplified using the appropriate primers listed in Table S6. These genes were amplified using the total genomic DNA of the SHPJ‐2 strain as a template. Furthermore, we synthesized two type V electron transfer chain components (*phtAcAd* from *M. vanbaalenii* PYR‐1 and *phdCD* from *Nocardioides* sp. KP7^17^) and one type III electron transfer chain component (*nahAaAb* from *Pseudomonas putida* NCIB 9816‐4^65^, ^66^). This allowed us to compare the degradation activities of various oxygenases. All synthesized genes were codon‐optimized with *E. coli* as the host and were amplified using the appropriate primers. The amplified genes encoding RHDs or P450s were then ligated with the plasmid pET‐28a(+) to generate oxygenase recombinant plasmids. Meanwhile, the genes encoding ferredoxin and ferredoxin reductase were ligated into the two multiple cloning sites of the plasmid pACYCDuet‐1. The resulting plasmids were electron transfer chain plasmids. The above‐mentioned plasmids, including oxygenase recombinant plasmids and electron transfer chain plasmids, were verified and subsequently cotransformed into the expression host *E. coli* BL21(DE3) for functional analysis. The *E. coli* BL21(DE3) strain transformed with the oxygenase plasmid was used as the control group.

### Overexpression of oxygenases and *in vivo* assays

*E. coli* BL21(DE3) containing recombinant plasmid(s) was cultured in an LB medium containing the appropriate antibiotics at 37°C. When OD_600_ reached 0.6–0.8, isopropyl‐β‐d‐thiogalactopyranoside with a final concentration of 0.2 mM was added to the culture and incubated at 16°C overnight. The cells induced overnight were collected by centrifugation, resuspended in phosphate‐buffered saline solution, and disrupted by a high‐pressure disruptor. SDS‐PAGE was used to examine the expression levels.

To confirm the specific function of oxygenases, the cells induced overnight were collected and prepared as resting cells with the OD_600_ of 5. After starving the cells without any substrate for 2 h, different substrates were supplemented into the culture and further incubated at 30°C for 24 h. The final concentration was 200 mg/l for PHE; 100 mg/l for ACE, NAP, DBF, DBT, FLN, CA, and indole; 5 mg/l for PYE, FLU, CHR, and BaA; 20 mg/l for ANT. The residual substrate concentrations were detected by HPLC at 24 h as mentioned above. Products catalyzed by various oxygenases were determined using the method described in Section “Determination of intermediate metabolites.”

## AUTHOR CONTRIBUTIONS

Lige Zhang designed the experiments; Lige Zhang and Huan Liu performed the experiments and analyzed the data; Lige Zhang and Hongzhi Tang wrote the manuscript; Lige Zhang, Huan Liu, Junbiao Dai, Hongzhi Tang, and Ping Xu revised the manuscript; Hongzhi Tang conceived the project.

## ETHICS STATEMENT

This study did not use animals.

## CONFLICT OF INTERESTS

The authors declare no conflict of interests.

## Supporting information

Supplementary Information.

## Data Availability

Raw data of metagenomic DNA of bacterial consortium PDMC were deposited in the NCBI Sequence Read Archive under the accession number SAMN21397100. The nucleotide sequences of the 16S rRNA gene of *Pseudomonas* sp. SHPJ‐1 and *Sphingobium* sp. SHPJ‐2 were deposited in NCBI under the accession numbers MZ433321 and MZ433319, respectively. The sequences of *phnA1B1*, *phnA2B2*, *orf4248‐orf4252*, and eight P450s genes of strain SHPJ‐2 were deposited into the NCBI under the accession numbers OL365393–OL365406.
