# Supplementary material for: Unveiling degradation mechanism of PAHs by a Sphingobium strain from a microbial consortium
Source: mLife. 2022 Jul 25;1(3):287–302. doi: 10.1002/mlf2.12032 (PMC10989954; doi:10.1002/mlf2.12032)
Supplement: Supplementary file 1 — Supplementary Information. [file MLF2-1-287-s001.doc]

# Supplementary Information

**Unveiling Degradation Mechanism of PAHs by a *Sphingobium* Strain from a Microbial Consortium**

Lige Zhang^1^, Huan Liu^1^, Junbiao Dai^2^, Ping Xu^1^ and Hongzhi Tang^1^*

^1^State Key Laboratory of Microbial Metabolism, Joint International Research Laboratory of Metabolic and Developmental Sciences, and School of Life Sciences and Biotechnology, Shanghai Jiao Tong University, Shanghai, People’s Republic of China

^2^CAS Key Laboratory of Quantitative Engineering Biology, Guangdong Provincial Key Laboratory of Synthetic Genomics and Shenzhen Key Laboratory of Synthetic Genomics, Shenzhen Institute of Synthetic Biology, Shenzhen Institutes of Advanced Technology, Chinese Academy of Sciences, Shenzhen, 518055, People’s Republic of China

*Corresponding author: H. Z. Tang; Mailing address: School of Life Sciences & Biotechnology, Shanghai Jiao Tong University, Shanghai 200240, P. R. China;

E-mail: [tanghongzhi@sjtu.edu.cn](mailto:tanghongzhi@sjtu.edu.cn); Tel: +86-21-34204066; Fax: +86-21-34206723


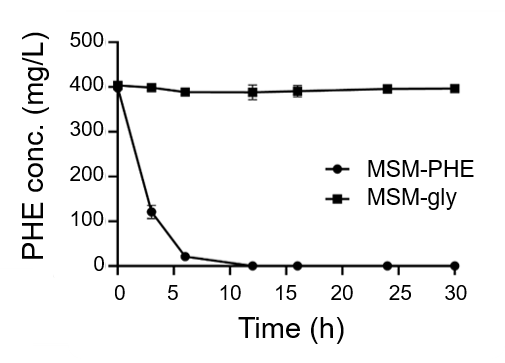


**Figure S1. Degradation of PHE by strain SHPJ-2 resting cells. “**MSM-PHE” and “MSM-gly” represent strain SHPJ-2 grown in MSM medium containing PHE or glycerol, respectively.

**
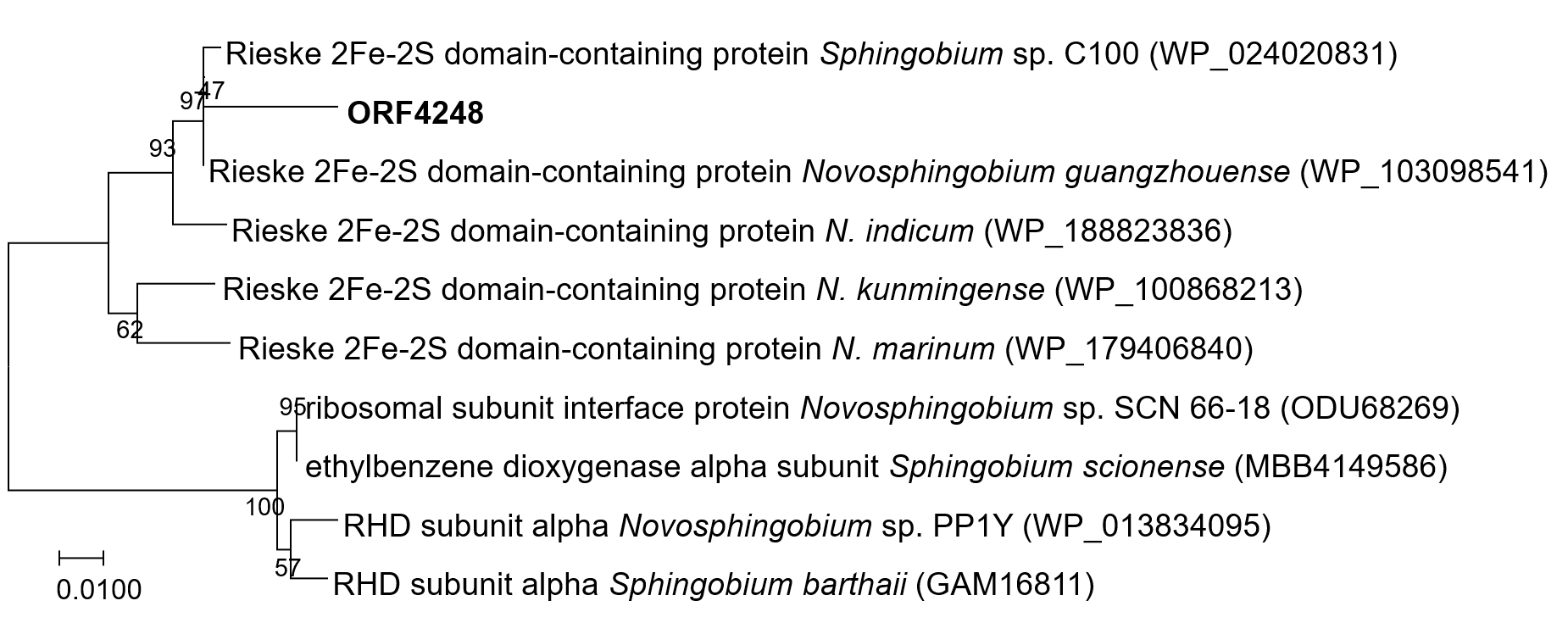
**

**Figure S2. Phylogenetic analysis of ORF4248**

**
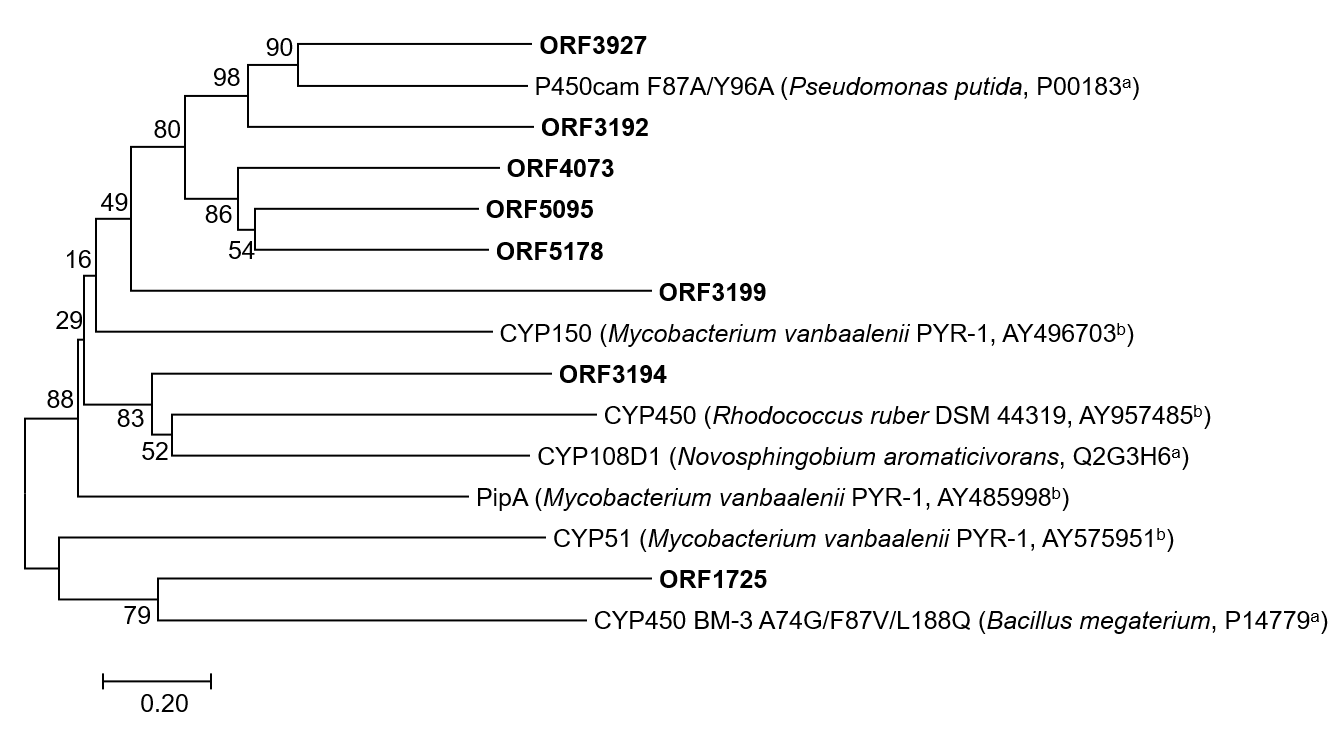
**

**Figure S3. Phylogenetic analysis of the P450s predicted in strain SHPJ-2 and the P450s that have been reported to degrade PAHs.** a indicates Uniprot number; b represents Genbank number.


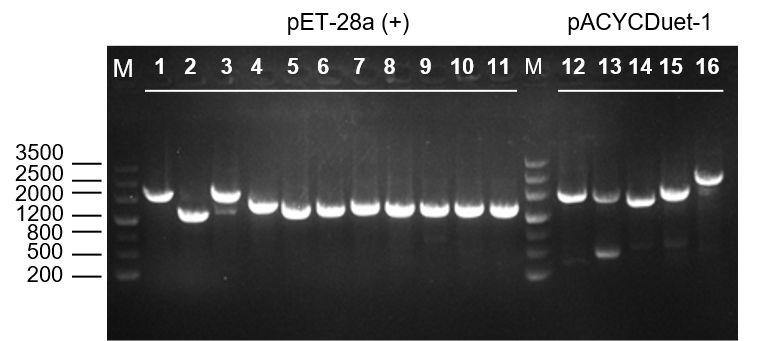


**Figure S4. PCR verification of the recombinant plasmid containing the core genes.** “M” represents DNA marker; different lanes represent PCR using different recombinant vectors as templates. Lanes 1 to 11 were pET-28a(+) recombinant vectors containing RHDs or P450s, and the carrying genes were *orf3136-3137, orf3890-3891, orf4249-4250, orf1725, orf3192, orf3194, orf3199, orf3927, orf4073, orf5095* and *orf5178*. Lanes 12 to 16 were pACYCDuet-1 recombinant vectors carrying the ETC coding genes *phtAcAd, phdCD, nahAcAd, orf4248-4252* and *orf5102-5105*.

**
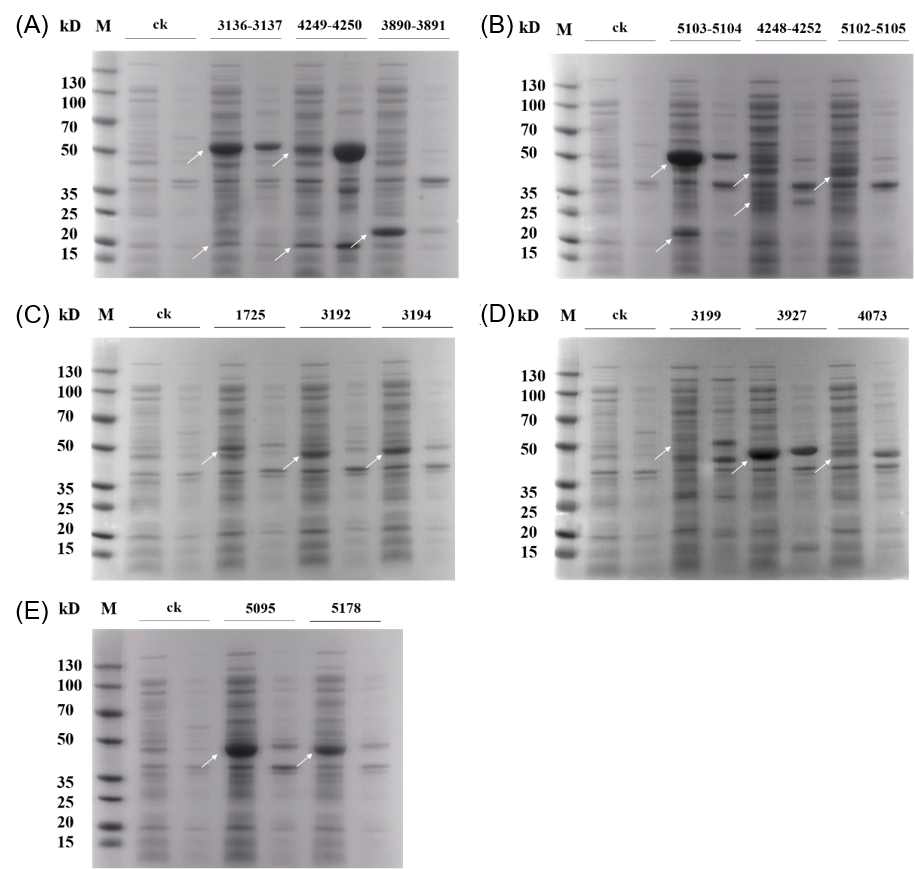
**

**Figure S5. SDS-PAGE analysis of functional proteins.** A~B are recombinant strains of RHDs and electron transfer chains; C~E are recombinant strains of P450s. “M” represents the protein marker; control and recombinant strains have two lanes, which are the supernatant sample (left) and the precipitation sample (right). The white arrow points to the expression band of the target protein.

**Table S1 Assembly splicing effect evaluation**

| **File** | **PDMC_contig.fa** |
| --- | --- |
| Min sequence length | 200 |
| Max sequence length | 984,922 |
| Total sequence number | 9,980 |
| N20 | 272,740 |
| N50 | 103,970 |
| N90 | 909 |
| N number | 0 |
| N rate | 0 |
| Total sequence length | 38,276,250 |
| GC number | 23,537,076 |
| GC content | 0.614926 |
| Sequences greater than 1kb | 2,036 |

**Table S2 PAHs and heterocyclic derivatives metabolic intermediates produced by the strain SHPJ-2**

| **PAHs** | | **Proposed products** | **RT (min)** | ***m/z* of fragment ions (ion abundances)** |
| --- | --- | --- | --- | --- |
| PHE | 1-Hydroxy-2-naphthoic acid | | 14.875 | 170(999),114(681),188(323),115(126),171(125) |
|  | 1-Naphthalenol | | 11.594 | 115(999),144(967),116(480),89(138),63(132) |
|  | Salicylic acid (TMS) | | 11.577 | 73(999),267(424),45(142),268(94),135(76) |
| NAP | 1-Naphthalenol | | 11.594 | 115(999),144(967),89(138),63(132),145(108) |
|  | 2-Naphthalenol | | 11.618 | 144(999),115(706),63(109),145(108),89(107) |
|  | 1,2-Dihydroxynaphthalene (2TMS) | | 14.251 | 304(999),73(760),305(281),216(171),186(137) |
|  | Salicylic acid (TMS) | | 11.577 | 73(999),267(424),45(142),268(94),135(76) |
|  | Coumarin | | 10.815 | 118(999),146(696),90(413),89(404),63(281) |
| ACE | 1-Acenaphthenone | | 13.541 | 168(999),140(858),139(757),69(155),169(139) |
|  | Aenaphthylene-1,2-dione | | 15.493 | 126(999),154(936),182(529),74(136),63(134) |
|  | 1*H*,3*H*-benzo[*de*]isochromen-1-one | | 14.817 | 155(999),127(705),184(661),126(217),183(197) |
|  | 1*H*,3*H*-benzo[*de*]isochromen-1,3-one | | 16.838 | 154(999),198(999),126(915),63(423),74(220) |
|  | 1,2-Dihydroxyacenaphthene (TMS) | | 16.711 | 328(999),73(620),329(302),45(123),330(106) |
| ANT | 9,10-Anthraquinone | | 16.284 | 152(999),180(947),208(862),151(527),76(463) |
| FLN | 1-Indanone | | 8.961 | 104(999),132(777),103(567),78(508),77(366) |
| FLU | Acenaphthylene-1,2-dione | | 15.493 | 126(999),154(936),182(529),74(136),63(134) |
|  | 1-Acenaphthenone | | 13.541 | 168(999),140(858),139(757),69(155),169(139) |
|  | 1,2-Dihydroxyacenaphthene (TMS) | | 16.711 | 328(999),73(620),329(302),45(123),330(106) |
| BaA | BaA-7,12-dione | | 21.493 | 258(999),101(551),202(484),100(402),230(362) |
| DBT | Dibenzothiophene-S-oxide | | 17.058 | 184(999),171(372),200(327),139(284),172(229) |
|  | Benzothiophene-2,3-dione | | 11.438 | 136(999),103(639),69(536),82(329),50(169) |
|  | Benzothiophene-2-ol | | 10.514 | 121(999),150(911),122(304),78(215),76(176) |
|  | 2-Mercaptobenzoic acid (TMS) | | 13.558 | 147(999),283(831),73(415),284(241),148(208) |
|  | Salicylic acid (TMS) | | 11.577 | 73(999),267(424),45(142),268(94),135(76) |
| DBF | 2-Hydroxy-2-(2’-hydroxyphenyl)acetate (3TMS) | | 13.148 | 267(999),73(334),268(242),179(102),269(97) |
|  | Salicylic acid (TMS) | | 11.577 | 73(999),267(424),45(142),268(94),135(76) |
| CA | 3-Hydrocarbazole (TMS) | | 16.007 | 239(999),224(583),73(522),166(162),45(138) |
| Indole | Anthranilate (TMS) | | 11.259 | 194(999),120(764),119(724),209(644),150(334) |

**Table S3 Degradation of PHE by heterologously expressed functional genes**

Oxygenase

ETC

|  | **Without ETC** | **5102-5105** | **4248-4252** | **PhdCD** | **PhtAcAd** | **NahAcAd** |
| --- | --- | --- | --- | --- | --- | --- |
| 3136-3137 | － | － | **++** | － | － | **+** |
| 3890-3891 | － | － | － | － | － | － |
| 4249-4250 | － | － | **++** | **+** | － | **++** |
| 5103-5104 | － | － | **+** | － | － | － |
| 1725 | － | － | － | － | － | **+++** |
| 3192 | － | － | **++** | － | － | － |
| 3194 | － | － | － | － | **+** | － |
| 3199 | － | － | **++** | － | － | － |
| 3927 | － | － | － | － | － | － |
| 4073 | － | － | **++** | － | － | **+++** |
| 5095 | － | － | **++** | － | － | **++** |
| 5178 | － | － | － | － | － | **+** |

+: 0% < degradation rate <30%; ++: 30% ≤ degradation rate < 50%; +++: degradation rate ≥ 50%; －: no degradation

**Table S4 Abbreviations**

| **Full name** | **Abbreviation** | **Full name** | **Abbreviation** |
| --- | --- | --- | --- |
| Polycyclic aromatic hydrocarbons | PAHs | Dibenzothiophene | DBT |
| Low-weight-molecular PAHs | LMW-PAHs | Carbazole | CA |
| High-weight-molecular PAHs | HMW-PAHs | Dibenzothiophene-S-oxide | DBTO |
| Phenanthrene | PHE | *N, N*-dimethylformamide | DMF |
| Naphthalene | NAP | *N,O*-bis(trimethylsilyl)trifluoroacetamide | BSTFA |
| Acenaphthene | ACE | Mineral salts medium | MSM |
| Fluorene | FLN | Phosphate-buffered saline | PBS |
| Anthracene | ANT | High-performance liquid chromatography | HPLC |
| Fluoranthene | FLU | Gas Chromatography-Mass Spectrometry | GC-MS |
| Benzo[a]anthracene | BaA | Electrospray ionization | ESI |
| Chrysene | CHR | Ring-hydroxylating dioxygenases | RHDs |
| Pyrene | PYE | Cytochrome P450 monooxygenases | P450s |
| Dibenzofuran | DBF | Metagenomic assembled genome | MAG |

**Table S5 Primers for RT-qPCR assays**

| **ORF** | **Sequence (5’-3’)** | |
| --- | --- | --- |
| 1725 | F: CGG CAA CAG CGT CTT CTC C | R: TGG GTC ATC ATC GGG TCG A |
| 3136 | F: GCT ATT TCG TTA CCA ATG TCG | R: TTG AAG CCG CTG TCT GTT |
| 3137 | F: TGC CGT TTG ACG ACC TTG | R: CCA GTG ACC GAC ATA GAG CC |
| 3192 | F: CCG CAG CGA CGA CAT CA | R: GACCACGGCAGGCATCA |
| 3194 | F: CAA TCC GCC ACT TAC CCG | R: TGC CCA TCA ATT TAT GAA CCA |
| 3199 | F: CGG CTA TGG CGT TCA TTT | R: CCA GGC GAC AGG CAG TT |
| 3890 | F: ACA AGA CCG AGC AGG TGA C | R: GTT GCG ATT GCG ATA AGC |
| 3891 | F: CGG GCA TTT CCA CCT TCC | R: CCT TTG GTC ACT TCC TCC TTG |
| 3927 | F: GCT TCA GGC CGC GAA TAA | R: TTG ACC ACC GTG TCC AGT CC |
| 4073 | F: CGC TGG GCG AAC GAA CT | R: TCG ACT GCA CCA GGA AGG AC |
| 4248 | F: ACA CCC GTC GGT TCG TCT | R: CAC CAA GCC CTT GTA GTT GC |
| 4249 | F: AGG TGC GGG TCC ATT CG | R: AAA GGC GTT GAG GTT GCT GT |
| 4250 | F: CGC AGG TCA ACA TAA CCA TT | R: AAG CCG AAG GTC AGG CAC |
| 4252 | F: CAA GCC GAC CGC ATC CT | R: TTC CTC GCC CAG CCA TT |
| 5102 | F: CCT ATC CTG TCG TCC TGC TGC | R: CTG CCA CTC CTG TCA TCT GCT |
| 5103 | F: CGA CGA CTG GGT GGC CTA T | R: CGT GCG GGA TGG TGG AT |
| 5104 | F: CCC GAA CAT TCT GAT AGC CT | R: CAG CGC CTG ATA TTT GAG C |
| 5105 | F: ATG CTG CGG TTA TGG CG | R: AAG GGC GGA CTG CGG ACA |
| 5153 | F: CGG TTT CGA TGA GTT GCT G | R: TGA ATA GTC GTT GCG GTG C |
| 5178 | F: TTG TGG AAT GAT GAC GAG GTG | R: TGC GAC GCC AGA TAG TTG A |
| 16S rRNA | F: CTG TAG TCG CCA TTG TAG CA | R: TGT CGT GAG ATG TTG GGT TA |

**Table S6 Primers for plasmid construction**

| **Primer** | **Sequence (5’-3’)** |
| --- | --- |
| 1725-F | AAGAAGGAGATATACCATGG CC acgcagcccttcaccccgccc |
| 1725-R | TGTCGACGGAGCTCGAATTC tcaaagccggacggcatcg |
| 3192-F | AAGAAGGAGATATACCATGG CC acaagcgtggctgaaggcg |
| 3192-R | TGTCGACGGAGCTCGAATTC tcagggccagcacaggtccag |
| 3194-F | AAGAAGGAGATATACCATGG CC gaacccgccgtcagaattc |
| 3194-R | TGTCGACGGAGCTCGAATTC tcagtcgccgaagcggacatg |
| 3199-F | AAGAAGGAGATATACCATGG CC acagaaatcaccagggcgaag |
| 3199-R | TGTCGACGGAGCTCGAATTC tcagtcccaggcgacaggcag |
| 3927-F | AAGAAGGAGATATACCATGG CC aacacggatacgctgacCC |
| 3927-R | TGTCGACGGAGCTCGAATTC tcaggctttccagacgagtgg |
| 4073-F | AAGAAGGAGATATACCATGG CC gagaccttcgctgcccgac |
| 4073-R | TGTCGACGGAGCTCGAATTC tcaggaccagtcgaggaccagat |
| 5095-F | AAGAAGGAGATATACCATGG CC atgtttcccgagcaaccgatc |
| 5095-R | TGTCGACGGAGCTCGAATTC tcagcttgcgggcgtccatac |
| 5178-F | AAGAAGGAGATATACCATGG CC acccaagtcgcacaacaatc |
| 5178-R | TGTCGACGGAGCTCGAATTC tcagccccattgcaggtggagg |
| 3890-3891-F | AAGAAGGAGATATACCATGGCC GTGTCGCAACCAGACAACTATT |
| 3890-3891-R | TGTCGACGGAGCTCGAATTC CTAAACGTAGATGTTGAGGTTC |
| 3136-3137-F | AAGAAGGAGATATACCATGG CC AATGGATCGTCGGCACTC |
| 3136-3137-R | TGTCGACGGAGCTCGAATTC TTATGCGAAGAAATAGAGATTC |
| 4249-4250-F | AAGAAGGAGATATACCATGG CC AACGACAACATTGCCGATCT |
| 4249-4250-R | TGTCGACGGAGCTCGAATTC TCAGAAAAAGGCGTTGAGGT |
| 5103-5104-F | AAGAAGGAGATATACCATGGCCATGCTGCACAATGCCGGGCTG |
| 5103-5104-R | TGTCGACGGAGCTCGAATTC TCAGAGGAAGATGGCGAAGTTG |
| 4248-F | aagaaggagatatacatatg cgcttcgaacggatcggtcg |
| 4248-R | gtttctttaccagactcgag ttacgccaatgatgtgtcgc |
| 4252-F | AATAAGGAGATATACCATGG CC ATGAAATCGGTAGCCGTGGTC |
| 4252-R | CCGAGCTCGAATTCGGATCC CTAACCAGCCTGCTTGAGG |
| 5102-F | aataaggagatataccatgg cc gtgacgggaaccccgcatatc |
| 5102-R | Ccgagctcgaattcggatcc tcacgctgccactcctgtcatc |
| 5105-F | aagaaggagatatacatatg agccagaaactgaaagtgg |
| 5105-R | gtttctttaccagactcgag tcaggcggctccgacggcaag |
| PhdCD-F | aataaggagatataccatgg cc CGTGTTGATGTGGACCCG |
| PhdCD-R | Ccgagctcgaattcggatcc TCAGGCGGTCGGGACGTCCG |
| PhtAcAd-F | aataaggagatataccatgg cc ggcggagttataaaggctaag |
| PhtAcAd-R | Ccgagctcgaattcggatcc ctatggtgatcgcgttgcggcg |
| nahAc-F | aataaggagatataccatgg cc GAACTGCTGATTCAGCCGAAC |
| nahAc-R | Ccgagctcgaattcggatcc TTAAATGCCGCCCGGATAAAAC |
| nahAd-F | aagaaggagatatacatatg ACCGTGAAATGGATTGAAG |
| nahAd-R | gtttctttaccagactcgag TTAGCTCAGATCAATCATCAC |
| T7 | TAATACGACTCACTATAGG |
| ACYCDuet up1 | GGATCTCGACGCTCTCCC |
| Duet down1 | GATTATGCGGCCGTGTACAA |
| Duet up2 | TTGTACACGGCCGCATAATC |
| T7 ter | TGCTAGTTATTGCTCAGCGG |

Underlined are restriction sites: CCATGG (*Nco*I), GAATTC (*Eco*RI), catatg (*Nde*I), CTCGAG (*Xho*I), GGATCC (*Bam*HI).
